# Mitochondrial DNA lineages determine tumor progression through T cell reactive oxygen signaling

**DOI:** 10.1073/pnas.2417252121

**Published:** 2025-01-03

**Authors:** Tal Yardeni, Arnold Z. Olali, Hsiao-Wen Chen, Liqing Wang, Jeffrey A. Halton, Angi Zenab, Ryan Morrow, Arrienne Butic, Deborah G. Murdock, Katrina G. Waymire, Grant R. MacGregor, Ben Boursi, Ulf H. Beier, Wayne W. Hancock, Douglas C. Wallace

**Affiliations:** ^a^Center for Mitochondrial and Epigenomic Medicine, The Children’s Hospital of Philadelphia, Philadelphia, PA 19104; ^b^Bert Strassburger Metabolic Center for Preventive Medicine, Sheba Medical Center, Tel Hashomer 5262000, Israel; ^c^Division of Transplant Immunology, Children’s Hospital of Philadelphia and Department of Pathology and Laboratory Medicine, University of Pennsylvania, Philadelphia, PA 19104; ^d^Division of Human Genetics, Department of Pediatrics, Perelman School of Medicine, University of Pennsylvania, Philadelphia, PA 19104; ^e^Department of Developmental and Cell Biology, Charlie Dunlop School of Biological Sciences, University of California, Irvine, CA 92697-2300; ^f^Division of Oncology, Sheba Medical Center, Tel-Hashomer, Tel-Aviv University, Tel Aviv 5262000, Israel; ^g^Center for Clinical Epidemiology and Biostatistics, Department of Biostatistics and Epidemiology, Perelman School of Medicine, University of Pennsylvania, Philadelphia, PA 19104; ^h^Immunology, Johnson & Johnson Innovative Medicine, Spring House, PA 19477

**Keywords:** cancer, mitochondrial DNA, immunotherapy, reactive oxygen species, adoptive transfer

## Abstract

Mitochondrial DNA (mtDNA) is highly polymorphic, and host mtDNA variation has been associated with altered cancer severity. To determine the basis of this mtDNA–cancer association, we analyzed conplastic mice with the C57BL/6J (B6) nucleus but two naturally occurring mtDNA lineages, *mtDNA^B6^* and *mtDNA^NZB^*, where *mtDNA^NZB^* mitochondria generate more oxidative phosphorylation (OXPHOS)-derived reactive oxygen species (mROS). In a cardiac transplant model, *mtDNA^B6^* Foxp3+ T regulatory (Treg) cells supported long-term allograft survival, whereas *mtDNA^NZB^* Treg cells failed to suppress host T effector (Teff) cells, leading to acute rejection. When challenged with melanoma or colon cancer cells, the *mtDNA^NZB^* mice exhibited strikingly impaired tumor growth while *mtDNA^B6^* mice showed Treg-dependent inhibition of Teff cells and allowed rapid tumor growth. Transcriptional analysis showed that activation of *mtDNA^NZB^* Teff cells increased mitochondrial gene expression while activation of *mtDNA^NZB^* Treg cells impaired mitochondrial gene expression and resulted in *mtDNA^NZB^* Treg cell exhaustion. Induction of the mitochondrially targeted catalytic antioxidant, mCAT, in hematopoietic cells normalized *mtDNA^NZB^* Treg function in both transplant and tumor models, indicating a key role for mROS in promoting Treg dysfunction. Anti-PD-L1 therapy did not modulate these effects, indicating that modulation of host mitochondrial function provides an independent approach for enhancing tumor cell destruction.

## Significance

Cancer is associated with reduced mitochondrial oxidative energy metabolism and naturally occurring variation in the maternally inherited mitochondrial DNA (mtDNA), which codes for critical components of mitochondrial energetics, can modulate tumor growth. mtDNA variation can modulate the immune profile through the oxidative metabolism of the anti-inflammatory T regulatory (Treg) cells, which limit antitumor effects of the more glycolytic inflammatory T-effector (Teff) cells. mtDNA variants that increase mitochondrial oxygen radical production can impair Treg cell function, releasing inhibition of the Teff cells to increase tumor destruction. Hence, mtDNA variation modulates tumorigenesis through immune cell metabolism, and modulating immune cell mitochondrial function may provide a alternative approach for cancer immunotherapy.

Since the observations of Otto Warburg that certain cancers manifest “aerobic glycolysis,” the reliance on glycolysis for biogenesis in the presence of oxygen (1), there has been interest in whether mtDNA variation might alter mitochondrial function and contribute to cancer development. There are three classes of clinically relevant mtDNA variation, ancient adaptive mutations, maternally inherited mutations, and de novo somatic mutations, all of which have been associated with in cancer (2, 3).

There are two ways that mtDNA variation might modulate tumorigenicity. Mutations in the tumor mtDNA could enhance tumorigenesis and in the process generate tumor antigens. Alternatively, host mtDNA variation might modulate the immune rejection of the tumor antigens.

Tumors can acquire mtDNA mutations that benefit tumor growth, but which generate tumor antigens that can be rejected by the immune system. These tumor-specific mutations commonly shift cancer cell metabolism from the predominately aerobic metabolism of a terminally differentiated cell back to the glycolysis characteristic of early development in which glycolytic substrates are conserved for cellular biogenesis (2–6). This may be the case for the differential fate of lung carcinoma cells harboring C57BL/6 (*mtDNA^B^*^6^) versus NZB (*mtDNA^NZB^*) mtDNAs (7). One way that de novo mtDNA variants can increase cancer cells’ growth is by inhibition of OXPHOS to increase mROS production which can serve as a cancer cell mitogen (8–10). Alternatively, OXPHOS inhibition could alter mitochondrial metabolites that serve as substrates for epigenomic modifying enzymes altering cancer cell gene expression (4, 5, 11).

Host mtDNA variation can affect tumorigenicity by modulating the host immune system. This has been documented in “mitochondrial-nuclear exchange (MNX)” mice in which the mouse mammary locus, PyMT on the FVB nuclear background, was combined with the FVB/NJ, C57BL/6J, and BALB/cJ mtDNAs. The different mtDNAs resulted in different tumor latencies with the C57BL/6J mtDNAs being longer and BALB/cJ mtDNAs being shorter, such that C57BL/6J mtDNA mice have less severe tumor burden and BALB/cJ mtDNA mice have greater tumor burden. The C57BL/6J mtDNA tumor cells were also found to have loosely coupled OXPHOS (12). Similarly, when mouse embryonic cell lines with C57BL/6 (B6) nuclei but bearing *mtDNA^B6^* versus *mtDNA^NZB^* mtDNA were injected into C57BL/6 mice with either *mtDNA^B6^* or *mtDNA^NZB^* mtDNAs, the tumors were rejected when the tumor and host mtDNAs were miss matched but not when the mtDNAs were matched (7).

**Fig. 1.**
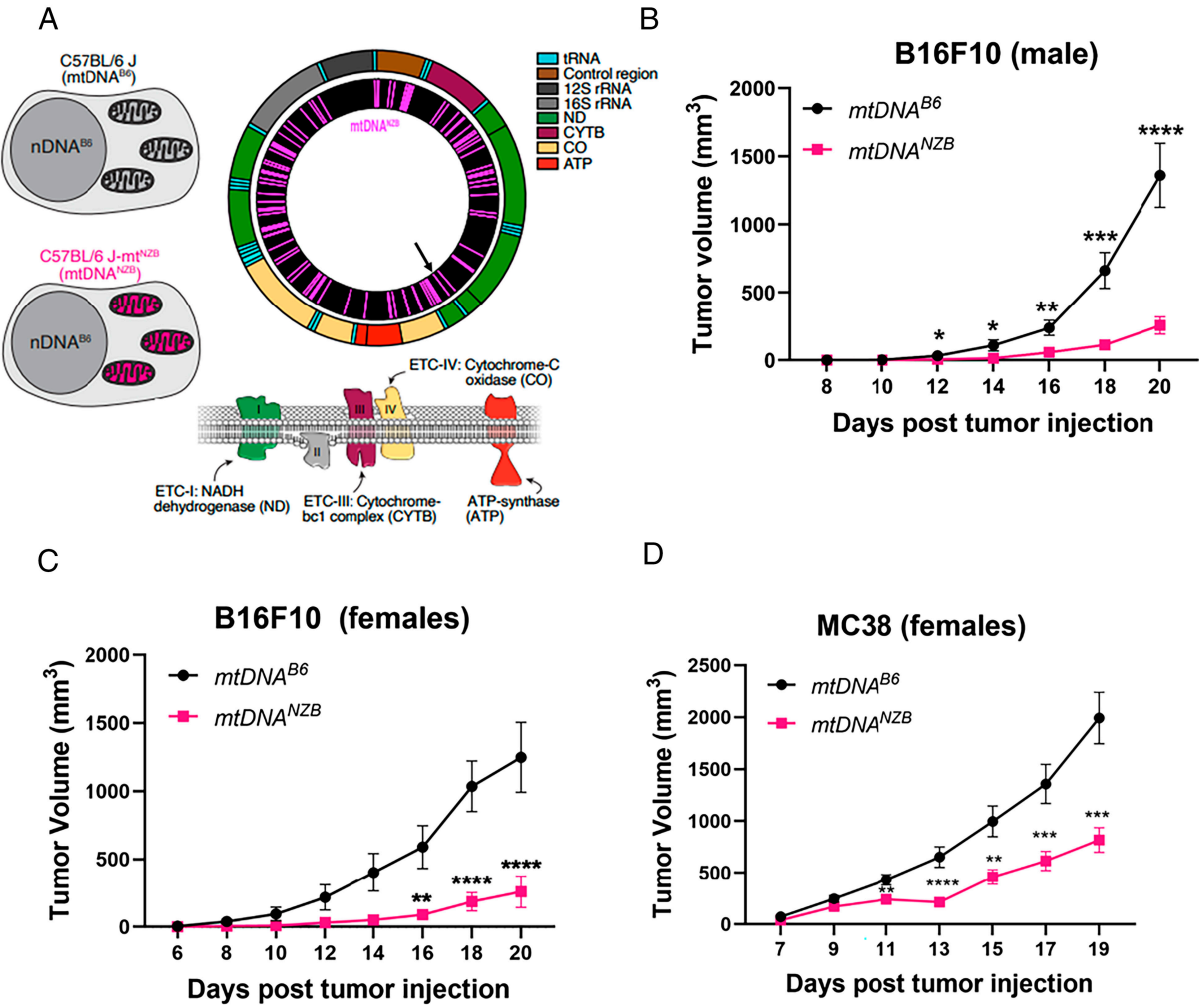
*mtDNA^B6^* and *mtDNA^NZB^* haplotypes affect tumor progression. (*A*) The two conplastic mouse strains with the same nDNA (C57BL/6J, B6) but differing only in their mtDNA haplotypes, *mtDNA^B6^* and *mtDNA^NZB^*. The *mtDNA^B6^* and *mtDNA^NZB^* mtDNAs differ at 91 nucleotides, the most important being the length of the homopolymer of A within the *tRNA^Arg^* DHU loop at nucleotide m.9821. The *mtDNA^NZB^ tRNA^Arg^* has 10 A while the *mtDNA^B6^* has 8 A; the extra *mtDNA^NZB^ tRNA^Arg^* DHU loop A results in increased mROS production. (*B* and *C*) B16F10 melanoma cells were injected into the flank of mtDNA male (*B*) and female (*C*) mice, and tumor growth was monitored. (*D*) MC38 colon adenocarcinoma cells were injected into the flank of mtDNA female mice. *mtDNA^B6^* (black, N ≥ 10 mice/experiment) and *mtDNA^NZB^* (pink, N ≥ 10 mice/experiment). *, **, ***, **** indicate *P* < 0.05, *P* < 0.01, *P* < 0.001, and *P* < 0.0001, respectively, two-way ANOVA test.

While tumor mROS production is important in tumor growth, it may also modulate host tumor rejection. The *mtDNA^B6^* and *mtDNA^NZB^* mtDNAs differ at 91 nucleotides and have different levels of mROS production in conplastic mice with the C57BL/6J nuclear background (13). The genetic variant that imparts this mROS difference has been proposed to be a polymorphism in mtDNA *tRNA^Arg^* gene at nucleotide m.9821 involving a homopolymer of adenosine (A) nucleotides, whose length ranges from 8 to 10 As. The *mtDNA^NZB^ tRNA^Arg^* has 10 As but the *mtDNA^B6^ tRNA^Arg^* has 8 As. The extra As have been proposed to increase mROS production, which in turn modulates mtDNA copy number to adjust OXPHOS to compensate for the reduced energetic function (14, 15) (Fig. 1*A*). B6 mice harboring *mtDNA^NZB^* versus *mtDNA^B6^* or the related *mtDNA^129^* (16) have different organ-specific phenotypes (17) and distinctive microbiomes (13). Mice that are heteroplasmic for the *mtDNA^NZB^* and *mtDNA^B6^/mtDNA^129^* mtDNAs have significant neurological deficits and directionally segregate the two mtDNAs in opposite directions in different tissues (18–20).

The phenotypic relevance of the heteroplasmic poly-A tract at the *tRNA^Arg^* locus is implicated in studies of the super-healing Murphy Roths Larger (MRL) mice which have striking wound regeneration capacity (21). These mice maintain a pseudohypoxic state resulting from the activation of the prolyl hydroxylase-hypoxia inducible factor (HIF-1α) pathway (22). Physiological analysis of this mouse has revealed increased aerobic glycolysis in association with mitochondrial clustering around the nucleus, increased mitochondrial content but partial OXPHOS dysfunction, and increased expression of the embryonic factors Nanog and Sox2. While the MRL fibroblasts have reduced ROS, quantified by DHR123 fluorescence, their glutathione levels are low in heart and fibroblasts suggesting that increased antioxidant defenses had reduced the overall cellular ROS levels (23). Surprisingly, the MRL mouse has a uniquely variable length heteroplasmic *tRNA^Arg^* poly-A tract with on average 2% 9As, 19% 10As, 35% 11As, 38% 12As, and 7% 13As. The most abundant *tRNA^Arg^* allele increases with age from 11As at 3 mo to 12As at 6 mo. As the adenosine nucleotides increase in number, the DHU loop size increases, but at 12As the structure of the *tRNA^Arg^* shifts pushing the extra As into the anticodon stem leaving only 5As in the DHU loop, a structure with one of the lowest ΔGs. Since the only two other mtDNA mutations in the MRL mtDNA were a heteroplasmic *tRNA^Met^* and a homoplasmic synonymous initiation codon variant in the *MT-ND3* gene it seems likely that the heteroplasmic *tRNA^Arg^* mutation plays an important role in modulating the MRL mouse phenotype (24).

While it is established that the host mtDNA background can modulate the foreign tumor growth, the mechanism by which the host rejects the foreign tumor antigens remains to be determined. Accordingly, we decided to analyze the host mechanism for rejecting various C57BL/6J tumors in our conplastic mouse strains harboring the B6 nucleus paired with *mtDNA^NZB^* or *mtDNA^B6^* mtDNAs (16). We found that mice with the *mtDNA^NZB^* mtDNA strongly impaired tumor growth relative to *mtDNA^B6^* mice and that this rejection occurred due to impaired T regulatory (Treg) cell function of the *mtDNA^NZB^* mice. This diminishes the constraints on the T effectors (Teff) cells, which then became more aggressive at rejecting the tumors. That this deficiency was linked to mROS production is consistent in our *MT-ND6^P25L^* mutant mouse with a partial OXPHOS complex I defect and increased mROS, the Treg cells of which decline with age. By contrast, Teff cells are more glycolytic, less affected by mitochondrial dysfunction, and thus are retained to aggressively attack the tumor cells (25).

## Results

### *mtDNA^B6^* and *mtDNA^NZB^* Haplotypes Modulate Tumor Progression.

Conplastic mice, sharing the same C57BL/6J (B6) nuclear DNA (nDNA) background but with mtDNAs originating from two different strains (C57BL/6J abbreviated as *mtDNA^B6^* and NZB abbreviated as *mtDNA^NZB^*) (Fig. 1*A*), exhibited significantly different capacities to sustain tumor growth. In *mtDNA^B6^* and *mtDNA^NZB^* mice injected subcutaneously with 0.1 × 10^6^ B16F10 melanoma cells (26), serial measurements showed that male and female *mtDNA^NZB^* mice had significantly reduced tumor volumes compared to *mtDNA^B6^* mice (Fig. 1 *B* and *C*). Reduced tumor progression in *mtDNA^NZB^* versus *mtDNA^B6^* mice was also observed upon challenge with MC38 colon adenocarcinoma cells (27) (Fig. 1*D*). Anti-PD-L1 immunotherapy did not significantly affect the growth of the B16F10 melanoma cells in the *mtDNA^B6^* and *mtDNA^NZB^* mice (*SI Appendix*, Fig. S1). Hence, mitochondrial haplotypes directly modulate solid tumor progression independent of immune checkpoint therapy.

### *mtDNA^B6^* and *mtDNA^NZB^* Haplotypes Regulate CD4^+^ Cell Function.

**Fig. 2.**
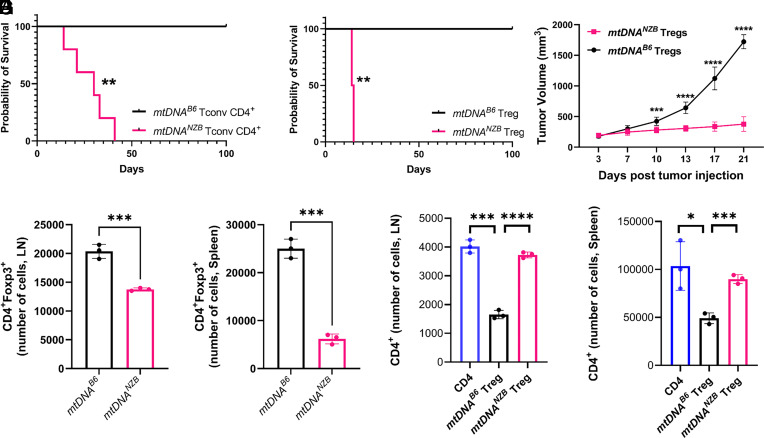
*mtDNA^B6^* and *mtDNA^NZB^* haplotypes regulate CD4^+^ cells function: disruptive effects of *mtDNA^NZB^* on T cell functions. (*A*) *mtDNA^NZB^* CD4^+^ conventional T cells (Tconv CD4^+^ which harbor a subset of Teff cells) were more resistant to Treg suppression than corresponding *mtDNA^B6^* Tconv CD4^+^ T cells, as shown by differing effects on allograft survival in *B6/Rag1*^−/−^ mice engrafted with BALB/c hearts and adoptively transferred with 0.5 × 10^6^ *mtDNA^B6^* Treg cells and 1 × 10^6^ *mtDNA^B6^* or *mtDNA^NZB^* Tconv CD4^+^ cells (Kaplan–Meier survival curve, **P < 0.01). (*B*) *mtDNA^NZB^* Treg cells lacked suppressive function compared to *mtDNA^B6^* Treg cells, as shown by differing effects on allograft survival in *B6/Rag1^−/−^* mice engrafted with BALB/c hearts and adoptively transferred with 0.5 × 10^6^ *mtDNA^B6^ or mtDNA^NZB^* Treg cells and 1 × 10^6^ *mtDNA^B6^* Tconv CD4^+^ cells (Kaplan–Meier survival curve, **P < 0.01). (*C*) *B6/Rag1^−/−^* mice were adoptively transferred with 1 × 10^6^ *mtDNA^B6^* CD4^+^Tconv cells plus 0.5 × 10^6^ *mtDNA^B6^* or *mtDNA^NZB^* Treg cells (10 mice/group) and 30 d later injected with 0.1 × 10^6^ B16F10 melanoma cells. Mean ± SEM. *P* values were determined by ordinary two-way ANOVA followed by Tukey’s multiple comparisons test (***P < 0.005; ****P < 0.001). (*D* and *E*) Treg cell survival was assessed by adoptive transfer of 0.5 × 10^6^ *mtDNA^B6^* or *mtDNA^NZB^* CD4^+^Foxp3^+^ Treg cells plus 0.1 × 10^6^ *mtDNA^B6^* Tconv CD4^+^ cells (4 mice/group) and the absolute numbers of Tregs determined by flow cytometry within (*D*) lymph nodes and (*E*) spleens 30 d later. Mean ± SEM. *P* values were determined by two-tailed unpaired *t* tests (***P < 0.005). (*F* and *G*) In homeostatic proliferation assays, congenic Thy1.1 + CD4^+^CD25^−^ *mtDNA^B6^* Tconv CD4^+^ cells were mixed in a 1:1 ratio with Thy1.2 + CD4^+^CD25^+^ Treg cells from *mtDNA^B6^* or *mtDNA^NZB^* donors, and adoptively transferred to *B6/Rag1^−/−^* mice (4 mice/group). After 7 d, the total number of Thy1.1 + CD4^+^ T cells in recipient (*F*) lymph nodes and (*G*) spleens was determined by flow cytometry. Mean ± SEM. *P* values were determined by ANOVA followed by Tukey’s multiple comparisons test (*P < 0.05, ***P < 0.005, and ****P < 0.001).

We previously showed that in a lactate-rich tumor microenvironment, oxidative Treg cells could prosper and suppress glucose-requiring Teff cells (28), leading us to investigate whether the antitumor phenotype of *mtDNA^NZB^* mice might result from differential Treg and Teff cell functions. We first compared baseline T cell numbers within the spleen, thymus, inguinal, or mesenteric lymph node cells (29, 30) but found no significant differences (*SI Appendix*, Fig. S2). To assess functional differences, we assessed the ability of adoptive transferred cells to reject cardiac allografts (31, 32). Treg cells (0.5 × 10^6^) from *mtDNA^B6^* mice were coadoptive transferred with 1 × 10^6^ CD4^+^ T cells from either *mtDNA^B6^* or *mtDNA^NZB^* mice into immunodeficient B6 recipients harboring BALB/c cardiac allografts. Recipients of *mtDNA^B6^* Tconv CD4^+^ cells exhibited long-term allograft survival (>100 d), but recipients of Tconv CD4^+^ cells from *mtDNA^NZB^* mice gradually rejected their grafts (*P* < 0.01) (Fig. 2*A*). Hence, Tconv CD4^+^ cells from *mtDNA^NZB^* mice have increased resistance to Treg suppression.

We then analyzed the capacity of adoptive transferred cells to reject BALB/c cardiac allografts immunodeficient B6 recipients by combining 1 × 10^6^ *mtDNA^B6^* Tconv CD4^+^ cells with 0.5 × 10^6^ Treg cells from either *mtDNA^B6^* or *mtDNA^NZB^* mice. This revealed that recipients of *mtDNA^B6^* Treg cells showed long-term allograft survival (>100 d), while recipients of *mtDNA^NZB^* Treg cells precipitously rejected the cardiac transplants (*P* < 0.01) (Fig. 2*B*). Hence, in transplant studies, Tconv CD4^+^ and Treg cells from *mtDNA^NZB^* mice showed altered immune responses with increased antigen rejection by Tconv CD4^+^ cells and reduced suppressive functions by Treg cells.

To confirm that this enhanced antigen rejection by B6 *mtDNA^NZB^* Treg cell function applied to a mouse tumor challenge, we injected 0.1 × 10^6^ B16F10 tumor cells into *Rag^−/−^* mice 1 mo after their adoptive transfer with 1 × 10^6^ Tconv CD4^+^ cells from *mtDNA^B6^* mice and 0.5 × 10^6^ Treg cells from either *mtDNA^B6^* or *mtDNA^NZB^* mice. Mice receiving *mtDNA^NZB^* Treg cells had markedly reduced B16F10 melanoma tumor growth compared to mice that received *mtDNA^B6^* Treg cells (Fig. 2*C*). Hence, the antitumor phenotype of *mtDNA^NZB^* mice can be attributed, at least in part, to reduced Treg function in *mtDNA^NZB^* mice.

### *mtDNA^NZB^* Mouse Treg Cells Exhibit Impaired Survival and Suppressive Function.

The demonstration of the increased Tconv CD4^+^ tumor suppressive phenotype in the presence of *mtDNA^NZB^* Treg cells could result from loss of Treg numbers, impaired Treg function, or both. To assess the effect of the *mtDNA^NZB^* on Treg survival, we adoptive transferred 0.5 × 10^6^ *mtDNA^B6^* or *mtDNA^NZB^* Treg cells and 0.1 × 10^6^ *mtDNA^B6^* Tconv CD4^+^ cells into *B6/Rag1^−/−^* mice. Thirty days after T cell engraftment, *mtDNA^NZB^* Treg cells were reduced in number when assessed in lymph nodes (LN) and spleens (Fig. 2 *D* and *E*). In addition, in an in vivo 7-d homeostatic proliferation assay, *mtDNA^NZB^* Tregs were unable to dampen T cell proliferation after adoptive transfer of 1 × 10^6^ CD4^+^ T cells and 0.5 × 10^6^ *mtDNA^B6^* or *mtDNA^NZB^* Treg cells into *B6/Rag1^−/−^* mice in LN and spleen (Fig. 2 *F* and *G*). Hence, compared to Treg cells from *mtDNA^B6^* mice, Treg cells from *mtDNA^NZB^* mice have impaired function and survival upon activation in vivo.

### *mtDNA^B6^* and *mtDNA^NZB^* Haplotypes Alter Gene Expression of Activated Tconv and Treg CD4^+^ Cells.

The adoptive transfer studies in cardiac transplant recipients revealed that both *mtDNA^NZB^* Tconv cells (Fig. 2*A*) and *mtDNA^NZB^* Treg cells (Fig. 2*B*) induced rejection, though the Treg cells caused a much more precipitous rejection than the Tconv cells. By contrast, both *mtDNA^B6^* Tconv and Treg cells induced tolerance.

**Fig. 3.**
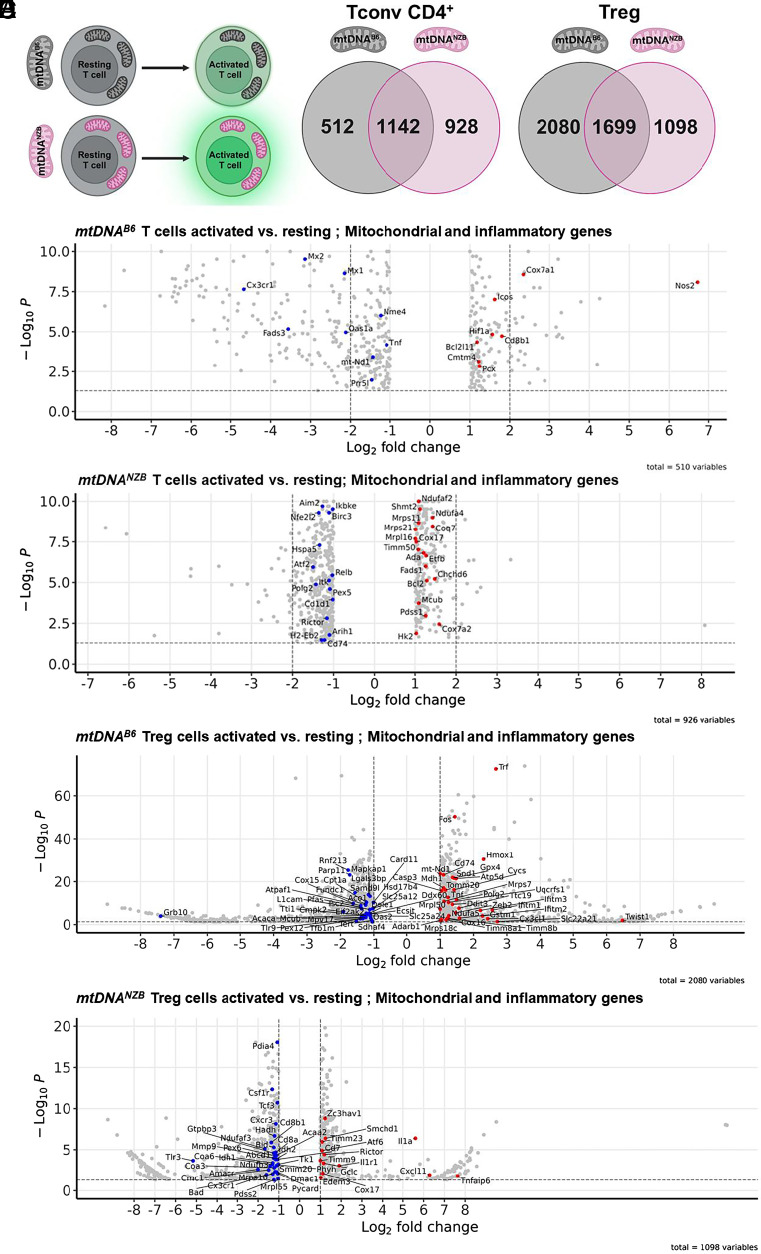
*mtDNA^B6^* and *mtDNA^NZB^* haplotypes modulate mitochondrial and inflammatory gene expression in activated Tconv and Treg CD4^+^ cells. (*A*) Tconv and Treg CD4^+^ cells isolated from either *mtDNA^B6^* or *mtDNA^NZB^* mice were activated with CD28/CD3 beads and the RNA isolated, converted to cDNA, and sequenced. The number of transcripts whose expression changed significantly up or down during T cell activation is indicated in overlapping Venn diagram. (*B*) Volcano plot of the unique genes significantly up- versus down-regulated in activated *mtDNA^B6^* Tconv CD4^+^ cells, with all mitochondrial and inflammatory genes labeled. (*C*) Volcano plot of the uniquely genes significantly up- versus down-regulated in activated *mtDNA^NZB^* Tconv CD4^+^ cells, with the mitochondrial and inflammatory genes whose expression changed more than twofold are labeled. (*D*) Volcano plot of the unique genes significantly up- versus down-regulated in activated *mtDNA^B6^* Treg cells, with all mitochondrial and inflammatory genes labeled. (*E*) Volcano plot of the unique genes significantly up- versus down-regulated in activated *mtDNA^NZB^* Treg cells, with all mitochondrial and inflammatory genes labeled (n = 3–5/ model). Red = up-regulated, blue = down-regulated.

To investigate both how and why the *B6 mtDNA^NZB^* Tconv and Treg CD4^+^ cells rejected the cardiac transplant, we performed RNA sequencing (RNAseq) on resting and activated Tconv and Treg CD4^+^ cells (33, 34). RNAseq revealed striking transcriptional differences in both the resting and activated Tconv and Treg cells of the *mtDNA^B6^* versus *mtDNA^NZB^* mouse cells. This is demonstrated by the number of up- and down-regulated genes seen in the isolated Tconv and Treg cells when analyzing the effects of antigen activation of resting T cells (Fig. 3*A*). For the Tconv cells, the expression of 1,142 gene changes was common to both *mtDNA^B6^* and *mtDNA^NZB^* mtDNAs, but 512 gene changes were unique for the activated *mtDNA^B6^* Tconv cells while 928 gene changes were unique for the *mtDNA^NZB^* Tconv cells. For the Treg cells, the expression of 1,699 gene changes was common to both *mtDNA^B6^* and *mtDNA^NZB^* mtDNAs, but 2,080 gene changes were unique for the activated *mtDNA^B6^* Treg cells but only 1,098 gene changes were unique for the *mtDNA^NZB^* Treg cells. Hence, the response of unique gene changes for *mtDNA^NZB^* Tconv cells was almost twice that of *mtDNA^B6^* Tconv cells, but the response of unique changes for *mtDNA^NZB^* Treg cells was half that of *mtDNA^B6^* cells (Fig. 3*A*).

#### Tconv cells: Unique gene changes for resting and activated *mtDNA*^*B6*^ versus *mtDNA*^*NZB*^ CD4+ Tconv cells.

Comparison of the transcriptional profiles of mitochondrial and inflammatory genes in resting versus activated *mtDNA^B6^* and *mtDNA^NZB^* Tconv cells showed marked differences. For both *mtDNA^B6^* or *mtDNA^NZB^* Tconv cells, activation increased mitochondrial gene expression and down-regulated inflammatory genes, but far fewer mitochondrial genes were affected in *mtDNA^B6^* Tconv cells than *mtDNA^NZB^* Tconv CD4^+^ cells (Fig. 3 *B* versus *C*) (33). Mitochondrial and bioenergetic genes that were uniquely up-regulated in activated *mtDNA^NZB^* CD4^+^ Tconv cells included (complex I-*Ndufaf2*, *Ndufa4*; complex IV-*Cox7a2*; mitoribiosome-*Mrps1*, *Mrpl16*; coenzyme Q synthesis-*Pdss1*, *Coq7*; protein import-*Timm50*; Fatty acid oxidation-*Etfb*, *Fads2*; calcium uniporter-*Mcub*; mitochondrial folate metabolism-*Shmt2*; antiapoptotic-Bcl2), and hexokinase 2 (*Hk2*) (Fig. 3*C*) (33). Among the uniquely up-regulated genes of the activated *mtDNA^B6^* Tconv CD4^+^ cells were *Hif1a* (hypoxia inducing factor-1α) which would increase glycolytic function, metabolic genes (*Pck*-pyruvate carboxylate, *Nos2*-mitochondrial nitric oxide synthase, NO inhibits complex IV), and immune genes (*Cmtm4*, *Cd8b*i, *Icos*, and *Bcl2l11*). Of particular note among the up-regulated *mtDNA^B6^* CD4^+^ Tconv cell mitochondrial genes was *Cox7a1* which has been implicated in OXPHOS complex IV dimerization (CIV_2_) (35) (Fig. 3*B*) (33). Thus, activation of *mtDNA^B6^* and *mtDNA^NZB^* Tconv CD4^+^ cells induced mitochondrial biogenesis, but activated *mtDNA^NZB^* Tconv CD4^+^ cells markedly increased the expression of mitochondrial genes relative to *mtDNA^B6^* Tconv CD4^+^ cells imparting different metabolic states.

Among the uniquely down-regulated genes in the activated *mtDNA^NZB^* Tconv CD4^+^ cells was mTORC1 (Fig. 3*C*). Among the uniquely down-regulated *mtDNA^B6^* Tconv CD4^+^ genes were inflammation genes (*Tnf*, *Mx1/2*, *Cx3cr1*, and *Oas1a*), nucleic acid synthesis genes (Pn5l and Nme4), and mtDNA genes (*MT-ND1*) (Fig. 3*B*).

To determine whether the tendency of *mtDNA^NZB^* cells to increase mROS production might influence the expression of antioxidant genes in the activated *mtDNA^NZB^* Tconv CD4^+^ cells, we surveyed changes in the expression of the known human–mammalian antioxidant genes (36). This revealed that activation of *mtDNA^NZB^* Tconv CD4^+^ cells induced 15 antioxidant genes and down-regulated 4, while activation of *mtDNA^B6^* Tconv CD4^+^ cells induced only 7 antioxidant genes and down-regulated 12 (*SI Appendix*, Fig. S3 *A* and B). Activated *mtDNA^NZB^* Tconv CD4^+^ antioxidant genes included the thioredoxin genes (*Txn2*, *Txnl1*, *Txndc9*, *Txndc12*, *Txndc17*, *Txnl4a*, and *Txn14D*) where thioredoxin participates in the reversal of oxidized cysteines and Txn2 is mitochondrially localized; Cu/Zn superoxide dismutase (*Sod1*), found in the mitochondrial intermembrane space and converts superoxide to hydrogen peroxide; Peroxiredoxin 2 (*Prdx2*) which reduces hydrogen peroxide and alkyl hydroperoxides; the glutaredoxin genes (*Glnx, Glnx3,* and *Glnx5*), which act in the antioxidant defense caused by reducing dehydroascorbate, peroxiredoxins, and methionine sulfoxide reductases; and protein disulfide isomerase (*Pdia6*) which catalyzes the oxidation-reduction and isomerization of disulfide bonds. The induction of these antioxidant genes, in particular mitochondrial *Txn2*, is consistent with activated *mtDNA^NZB^* Tconv CD4^+^ cells experiencing greater oxidative stress than the *mtDNA^B6^* Tconv CD4^+^ cells (*SI Appendix*, Fig. S3 *A* and B).

#### Treg cells: Unique gene changes for resting and activated *mtDNA^B6^* versus *mtDNA^NZB^* CD4^+^ Treg cells.

Unique changes following activation of isolated *mtDNA^B6^* and *mtDNA^NZB^* Treg cells revealed that both mtDNA genotypes modulated mitochondrial and inflammatory gene expression (34). However, the response of the *mtDNA^B6^* Treg cells to activation was much more robust than that of the *mtDNA^NZB^* Treg cells. This supports the conclusion that the *mtDNA^NZB^* Treg cells are severely impaired in their response to antigen activation (Fig. 3 *D* and *E*). Focusing on the *mtDNA^NZB^* Treg cells revealed over 20 key mitochondrial and inflammatory genes being up-regulated in the resting *mtDNA^NZB^* Treg cells compared to *mtDNA^B6^* Treg cells. These genes included *Cd74*, *Hmox1*, *Trf*, *Clita*, *Cd8a*, *Cd8b1*, *Ifitm3*, *Prr5*, *Il1b*, *Lrrk2*, *Zeb2*, *Ifitm1*, *Ifitm2*, *Plaur*, *Nubpl*, *Cxcl9*, *Nlrp3*, *Tlr3*, *Cx3cr1*, *H2*-*Eb2*, *Slc22a21*, and *Csf1r* (*SI Appendix*, Fig. S3*C*). The differential expression of these genes in resting *mtDNA^NZB^* Treg cells is indicative of immune exhaustion, mitochondrial dysfunction, and improper regulation of immune responses during stress. This could explain the dysfunction of the activated Treg from *mtDNA^NZB^* mice. The upregulation of *Ifitim3* and *Zeb2* are linked to T cell exhaustion while the upregulation of *Il1b*, *Nlrp3,* and *Lrrk2* genes involved in inflammasome activation suggests a heightened proinflammatory state which upon overactivation could trigger activation-induced cell death. The *Hmox1* gene is involved in cellular stress responses while *Nubpl* is important in mitochondrial biogenesis suggesting that oxidative and mitochondrial stress could play a role in Treg dysfunction, T cell exhaustion, and death during activation (37, 38). Upregulation of chemokine-related genes like *Cxcl9* and *Cxclcr1* play crucial roles in Treg trafficking and could exacerbate the response to inflammatory environments leading to stress-induced cell death during activation (39, 40). The altered expression of *mtDNA^NZB^* Tregs genes such as *Nubpl* for mitochondrial function and *Lrrk2* for inflammation thus point toward energy deficits and immune regulation imbalances as impairing Treg cell function following activation (*SI Appendix*, Fig. S3*C*).

Upon activation, we observed the differential gene induction in *mtDNA^NZB^* Treg cells for mTORC1 (*Rictor*), mitochondrial assembly (*Timm9*, *Timm23*, and *Cox17*), inflammation (*IL1α*, *IL1r1*, *Cd7*, *Cxcl11*, and *Tnfaip6*), DNA and viral response (*Zc3hav1* and *Smchd1*), mitochondria–ER interaction (*Edem1* and *Atf6*), and the glutathione antioxidant synthesis (*Gclc*) (Fig. 3*E*). The down-regulated genes include multiple mitochondrial and inflammatory genes. Mitochondrial and peroxisomal genes included (*Nudfb2*, *Ndufaf3*, *Cmc1*, *Coa3*, *Coa6*, *Smim20*, *Hadh*, *Amacr*, *Acaa2*, *Pdss2*, *Mrpl55*, *Idh2*, and *Pex6*), mitochondrial proapoptotic genes (*Bad* and *Bid*), inflammation genes (*Csfir*, *Tcf3*, *Cxcr3*, *Cd8b1*, *Cd8a*, *Dmac1*, *Pycad*, and *Cxcr1*), and the cellular biogenesis and metabolism genes (*Idh1*, *Pdia4*, *Aca2*, *TK1*, *Mrpa1*, and *Tir3*) (Fig. 3*E*). Hence, upon activation, *mtDNA^NZB^* Treg cells show a striking downregulation of mitochondrial function and impaired inflammatory and biogenesis genes consistent with bioenergetic failure. Finally, activated *mtDNA^B6^* Treg cells induced the antioxidant genes (*Sod1*, *Gpx4*, *Txndc17*, and *Prdx1*), which were not induced in *mtDNA^NZB^* Treg cells (*SI Appendix*, Fig. S3 *D* and E).

To validate the effects of Treg activation between the *mtDNA^NZB^* and *mtDNA^B6^* Treg cells, we performed RT-qPCR quantification on selected genes. While levels of *Foxp3* remained comparable between *mtDNA^NZB^* and *mtDNA^B6^* Treg cells, *mtDNA^NZB^* Tregs at rest had higher levels of several Treg-associated genes (*CD25, GITR,* and *TGFb1*) than *mtDNA^B6^* Treg cells, and these differences were also seen upon 2 h of activation (*SI Appendix*, Fig. S4). Surprisingly, however, activation of *mtDNA^NZB^* Treg cells promoted the elaboration of abnormally higher levels of proinflammatory cytokines (e.g., *IFN-γ, IL-2*) than *mtDNA^B6^* Treg cells (*SI Appendix*, Fig. S4 *E* and F) indicative of dysfunctional *mtDNA^NZB^* Treg cells (41–44).

Thus, transcriptional analysis of activated *mtDNA^NZB^* Tconv CD4^+^ versus *mtDNA^B6^* Tconv CD4^+^ cells revealed that the *mtDNA^NZB^* results in the induction of mitochondrial genes, but downregulation of inflammatory genes. These changes could be indicative of enhanced innate immune activation. By contrast, *mtDNA^NZB^* Treg CD4^+^ versus *mtDNA^B6^* Treg CD4^+^ cells manifest a decline in mitochondrial function and evidence of Treg cell exhaustion indicative of Treg cell failure and enhanced adaptive immune inflammation.

### Decreased *mtDNA^NZB^* mROS in Immune Cells Reduces Antitumor Immune Response.

**Fig. 4.**
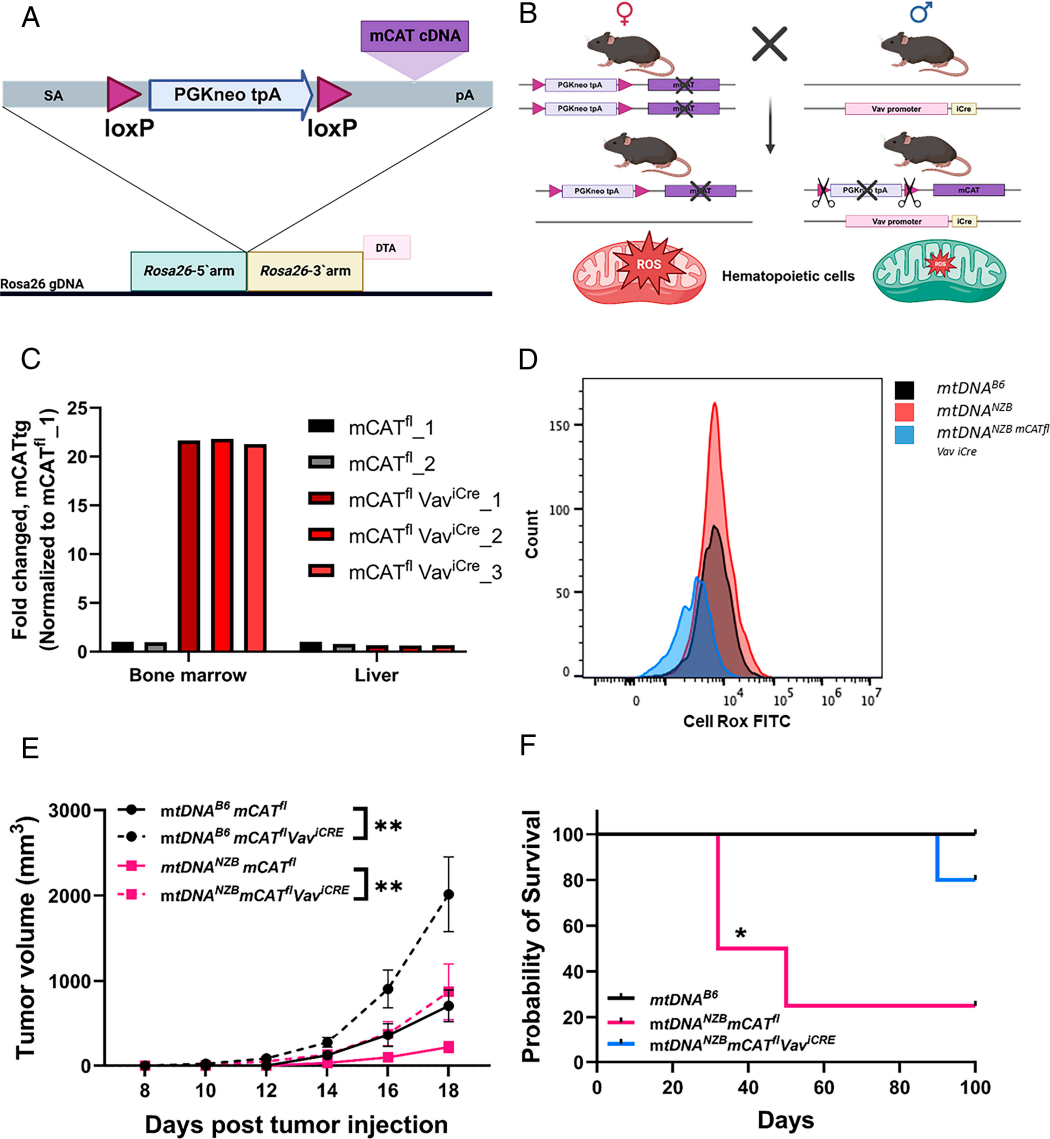
Decreased ROS in *mtDNA^B6^* and *mtDNA^NZB^* immune cells reduces antitumor immune responses. (*A*) Generation of Rosa26-LSL-mCAT (*mCAT^fl^*) allele. Knock-in of splice-acceptor (SA) *mCAT* cDNA downstream of the *Rosa26* constitutive promoter separated by a LoxP-STOP-LoxP (LSL) sequence. (*B*) Breeding strategy for *mCAT^fl^* with *Vav^iCre^* mice. (*C*) Induction of *mCAT* in mouse tissues when *mCAT^fl^* was paired with *Vav^iCre^.* In the hematopoietic cells *mCAT* induction resulted in ~21-fold increase of *mCAT* mRNA only in the bone marrow (BM) and not in the liver when compared to the control littermates (*mCAT^fl^* without *Vav^iCre^*). (*D*) Levels of ROS production detected by flow cytometry in resting and (CD3/CD28 beads, 2 h) activated *mtDNA^B6^*, *mtDNA^NZB^*, or *mtDNA^NZB^ mCAT^fl^ Vav^iCre^* CD4^+^ Tconv cells assayed using a CellRox Green. (*E*) Reduced mROS in the hemopoietic cells increases melanoma tumor volumes. B16F10 melanoma cells (0.1 × 10^6^) were injected subcutaneously into *mtDNA^B6^* (black) and *mtDNA^NZB^* (pink). Solid line – Control (*mCAT^fl^* not expressed), dash line mROS reduced (*mCAT^fl^, Vav^iCre^*), N > 10/group and repeated twice two-way ANOVA test, **P < 0.01. (*F*). Differential rejection of BALB/c (H-2^d^) cardiac allografts in immunocompetent *mtDNA^B6^*, *mtDNA^NZB^*, or *mtDNA^NZB^ mCAT*^fl^ *Vav^iCre^* allograft recipients that were treated with CD154 mAb/DST at the time of engraftment; in contrast to *mtDNA^NZB^* recipients that rejected their grafts, *mtDNA^NZB^* recipients with the activated mCAT (*mtDNA^NZB^*, *mCAT^fl^, Vav^iCre^*) sustained graft survival, Kaplan–Meier survival curve, *P < 0.05.

To confirm that the increased tumor rejection was due to mROS damage to the *mtDNA^NZB^* Treg cells, we generated a mouse strain that expresses cre-recombinase conditional mitochondrial targeted catalytic antioxidant catalase (mCAT) from within the *Rosa26* locus (Rosa26-LSL-mCAT or *mCAT^fl^*) (Fig. 4*A*; *SI Appendix*, Fig. S5 and Dataset S1). *mCAT^fl^* homozygous females were crossed with males heterozygous for a hematopoietic tissue-specific Cre (*Vav^iCre^*), resulting in all F1 offspring having one allele of the *mCAT^fl^* transgene and half of the offspring also inheriting the *Vav^iCre^* transgene thus activating the *mCAT* transgene in their hematopoietic cells (Fig. 4*B*). In the *mCAT^fl^ Vav^iCre^* mice, catalase mRNA increased approximately 21-fold in the bone marrow but not in the liver (Fig. 4*C*), and ROS was decreased in the bone marrow cells of the *mtDNA^NZB^* mice without affecting respiration (*SI Appendix*, Fig. S6). Compared to *mtDNA^B6^* mice, mROS levels were increased in *mtDNA^NZB^* Tconv CD4^+^ cells, and induction of mCAT reduced mROS levels (Fig. 4*D*). In *mtDNA^B6^* and *mtDNA^NZB^* mice in which the *mCAT^fl^* transgene was induced in hematopoietic cells (*mCAT^fl^ Vav^iCre^*) we found a marked increase in B16F10 melanoma tumor growth (Fig. 4*E*). Likewise, the *mtDNA^NZB^* mice expressing mCAT in their hematopoietic cells no longer could reject a cardiac allograft when treated with a tolerance-inducing protocol of CD154 mAb (MR-1) plus donor splenocyte transfusion (*P* < 0.01) (Fig. 4*F*).

## Discussion

While it has been observed that ancient host mtDNA haplotype variants are associated with differential tumorigenicity (7, 12), the mechanism by which the host mtDNA genotype limits tumor progression remains to be clarified. To address this gap, we have demonstrated that while C57BL/6J (B6) mice with B6 nuclei and B6 mtDNA (*mtDNA^B6^*) are permissive for melanoma and colon cancer tumor propagation, the conplastic mice with B6 nuclei but NZB mtDNA (*mtDNA^NZB^*) strongly suppresses tumor cell growth.

Previously, we showed that mice harboring the mtDNA *ND6^P25L^* missense mutation have increased mROS production and reduced T regulatory (Treg) cells (25) implying that mtDNA variation could reduce Treg suppression of Teff cell cytotoxicity making the adaptive immune system more aggressive at rejecting foreign antigens. Since it has been established that conplastic B6 mice with *mtDNA^NZB^* have greater mROS production than B6 mice with *mtDNA^B6^*, presumably due to the increased number of the As in the *tRNA^Arg^* DHU loop (13–15, 24), we hypothesized that B6 *mtDNA^NZB^* mice would have increased mROS, reduced Tregs, and increased Teff cell function resulting in more aggressive Teff cell attack of foreign antigens, tumor or cardiac.

To determine whether the B6 *mtDNA^NZB^* Treg cells are impaired in their ability to limit Teff cell function, we employed adoptive transfer of *mtDNA^B6^* versus *mtDNA^NZB^* Treg and Teff cells in a cardiac allograft rejection model. This revealed that both the B6 *mtDNA^NZB^* Tconv cells, harboring the Teff cells, as well as the Treg cells rejected the heart allograft while the B6 *mtDNA^B6^* Tconv and Treg cells did not. The cardiac allograft rejection by the B6 *mtDNA^NZB^* Treg cells was more aggressive than that of the B6 *mtDNA^NZB^* Tconv cells which confirmed that the adaptive immune system of the B6 *mtDNA^B6^* and B6 *mtDNA^NZB^* mice was central to Treg suppressiveness and thus Teff cell rejection of foreign antigens.

Though less precipitously than the adoptive transfer of the B6 *mtDNA^NZB^* Treg cells, B6 *mtDNA^NZB^* Tconv cells also caused rejection of the cardiac allograft. This indicates that in addition to Treg cell adaptive immune function, a second immune function was contributing to allograft and tumor rejection. Since the B6 *mtDNA^NZB^* Tconv cells contain Teff cells but also other immune cells, Tconv cell antigenic rejection could be due to enhanced function of the resident innate immune cells. Increased mROS would augment the release of mtDNA through the mitochondrial permeability transition pore to activate the innate immune systems (45). This could account for the previous report that inactivation of NK cells permitted *mtDNA^B6^* carcinoma growth in B6 *mtDNA^NZB^* mice, though requiring twice as long for the tumors to form (7).

To determine the differential metabolic and immune effects of the B6 *mtDNA^B6^* and B6 *mtDNA^NZB^* Tconv and Treg cells, we performed RNA sequencing of resting and activated B6 *mtDNA^B6^* and B6 *mtDNA^NZB^* Tconv and Treg cells. The B6 *mtDNA^B6^* and B6 *mtDNA^NZB^* Tconv and Treg cells were found to differ at rest as well as upon activation and that activated B6 *mtDNA^NZB^* Treg cells were immunologically defective.

To establish that the differential B6 *mtDNA^B6^* and B6 *mtDNA^NZB^* immune functions were related to increased mROS we created an inducible mitochondrially targeted catalase (mCAT). When mCAT was induced in the hematopoietic cell lineage of the B6 *mtDNA^NZB^* Treg cells this suppressed both cardiac allograft and tumor rejection. Hence, the increased *mtDNA^NZB^* mROS impairs B6 *mtDNA^NZB^* Treg function reducing suppression of Teff cell function and enhancing innate immune function resulting in the increased destruction of both the cancer cells and cardiac allografts.

Our interpretation of present and the previous results is that mtDNA functional variation can modulate both the adaptive immune system through modulation of Treg cell suppressiveness and the innate immune system through increasing aggressiveness of the Tconv cells. This leads to an interesting dichotomy. Inhibiting mROS should reduce the mitogenic effects of mROS on tumor growth, but reduction in mROS could also moderate both the adaptive and innate immune systems compromising the immune rejection of the tumor. This tradeoff demonstrates how subtle changes in mtDNA sequence and function can have profound effects on the regional adaptability of human populations (46).

## Materials and Methods

### Mice.

Institutional Animal Care and Use Committee’s from the Children’s Hospital of Philadelphia and Sheba Medical Center (SMC) approved all animal protocols in this research. All mice were maintained on a 13-h/11-h light–dark cycle. Most of the experiments were performed on 2- to 3-mo-old males, with part of the tumor inocluation experiments and the adoptive transfer experiments performed on females. The conplastic mice were bred and maintained in the Wallace laboratory colony for more than 20 y and in the Yardeni (SMC) laboratory for over 2 y. These included two conplastic mouse strains, encompassing C57BL/6J (B6) nDNA but with either *mtDNA^B6^* or *mtDNA^NZB^* mtDNAs. *Rag1^−/−^* mice (JAX #002216) were used for the adoptive cell transfer experiments.

We also generated a mouse strain that expresses a conditional mitochondrial targeted catalytic antioxidant catalase (mCAT) following cre-recombinase expression, from the *Rosa26* locus (Rosa26-LSL-mCAT or *mCAT^fl^*). The mCAT cDNA (47) was knocked into the constitutively active *Rosa26* locus downstream from a loxP-STOP-loxP (LSL) sequence. The plasmid construct harbored the *Rosa26* 5′ arm, an RNA splice acceptor (SA) sequence, a Lox P site, a PGK1 promoter and *Neo* gene, the PGK-1 3′ UTR and polyA termination signal, three SV40 poly A sites, a second Lox P site, the human catalase cDNA with its 5′ ornithine transcarbamylase (OCT) mitochondrial targeting peptide, the bovine growth hormone poly A site, and the *Rosa26* 3′arm. A diphtheria toxin A expression cassette was included after the 3′ Rosa26 arm to select against random integration of the targeting construct. The *Rosa26-LSL-mCAT* plasmid map and the annotated plasmid sequence are provided in See *SI Appendix*, Fig. S5 and Dataset S1. The *Rosa26-LSL-mCAT* cassette was linearized and then electroporated into mouse JM8.N4 (C57BL/6NTac) embryonic stem cells. ES cells containing the targeting construct were selected with G418, and clones with homologous insertion confirmed by restriction analysis followed by Southern blotting using probes outside the targeting construct. The *Rosa26-LSL-mCAT* allele was backcrossed on to a C57BL/6J nuclear background. When this transgene was bred with Vav^iCre^ mice (JAX #008610) mCAT was induced in the hematopoietic cells (Fig. 4 *B* and *C*).

### Tumor Inoculations.

Two tumor cell lines were used in this study: B16F10 melanoma (26), and MC38 colon adenocarcinoma (27). Both cell lines were derived from C57BL/6J mice. The cell lines were grown in RPMI 1640, 10% FBS, 2 mM glutamine, and 5 μg/mL penicillin and streptomycin. For injection, 0.1 × 10^6^ (B16F10), and 2 × 10^6^ (MC38) cells were suspended 100 μL phosphate-buffered saline (PBS) and injected subcutaneously into the left flank. After 6 d posttumor inoculation, the tumor size was measured every other day until the first mouse reached to the end point of 15 mm of the longest diameter of tumor. *Rag1^−/−^* mice were adoptively transferred with 1 × 10^6^ *mtDNA^B6^* Tconv CD4^+^ cells plus 0.5 × 10^6^ *mtDNA^B6^* or *mtDNA^NZB^* Treg cells (10 mice/group), and 30 d later were injected with 0.1 × 10^6^ B16F10 melanoma cells. Tumor volumes were determined twice/week for 3 wk using the formula (3.14 × long axis × short axis × short axis/6).

### Immunotherapy Treatment.

Following the inoculation of B16F10 melanoma cells, the mice were divided into two groups. The first group received intraperitoneal injections of 100 µg of anti-PD-L1 on days 7, 10, 13, and 16 posttumor inoculation, while the second group was injected with saline as a control (48).

### Spleen and Lymph Node Immune Cell Distribution.

Murine spleen, inguinal and mesenteric peripheral lymph nodes, and thymus were harvested and processed to obtain single-cell suspensions of lymphocytes. Red blood cells were removed by hypotonic lysis. The cells were stained with antibodies for flow cytometry staining (49), suspended in PBS with 2% FBS (Thermo Fisher Scientific Cat# 16000044). The antibodies used were CD4 Pacific Blue (Clone RM4-5, BD Biosciences, RRID:AB_397030), CD8 FITC (Bio-Rad, RRID:AB_2229098), CD44 PE (BD Biosciences, RRID:AB_394649), CD62L APC-Cy7 (RRID:AB_2795170), CD25 APC (Clone PC61, BD Biosciences, RRID:AB_398623), All flow cytometry data were captured using Cytoflex (Beckman Coulter, Brea, CA) and analyzed using the FlowJo 10.10 software.

### T Cell Isolation.

Splenocytes were extracted from mice and T cells were isolated using magnetic-activated cell sorting (MACS). The Treg (CD4^+^ CD25^+^) and Tconv CD4^+^ (CD4^+^ CD25^−^) cells were isolated in two steps. First, the splenocytes were reacted with magnetic-bound antibodies to negatively select for CD4^+^ cells (130-104-454, Miltenyi), as well as with PE-conjugated CD25 antibodies (130-091-041, Miltenyi). The flow through from the MACS column was enriched for CD4^+^ cells and was verified by flow cytometry for the enrichment of CD4^+^ cells (*SI Appendix*, Fig. S7). Then, the PE-conjugated anti-CD25 antibody cells were selected using anti-PE conjugated magnetic beads. This resulted in the flow through being Tconv CD4^+^ (CD4^+^ CD25^−^) cells and the bound cells being Treg (CD4^+^ CD25^+^) cells. Flow cytometric analysis of aliquots of the latter cells showed they were >95% Foxp3^+^.

### Cardiac Allografts.

BALB/c (H-2^d^) hearts were transplanted heterotopically into the abdomens of C57BL/6 (H-2^b^) *Rag1^−/−^* of *mtDNA^B6^*, *mtDNA^NZB^*, or *mtDNA^NZB^ mCAT*^fl^ *Vav^iCre^* recipients, all on the B6 background, and graft survival was monitored daily by palpation and confirmed by histology (50). In reductionist studies, immunodeficient *Rag1^−/−^* allograft recipients were adoptively transferred, at the time of transplantation, with 1 × 10^6^ *mtDNA^B6^* or *mtDNA^NZB^* Tconv CD4^+^ cells plus 0.5 × 10^6^ *mtDNA^B6^* Treg cells, or with 1 × 10^6^ *mtDNA^B6^* Tconv CD4^+^ cells plus 0.5 × 10^6^ *mtDNA^B6^* or *mtDNA^NZB^* Treg cells. Subsequently, immunocompetent *mtDNA^B6^*, *mtDNA^NZB^*, or *mtDNA^NZB^ mCAT*^fl^ *Vav^iCre^* allograft recipients were treated with CD154 mAb (250 µg, i.v.) plus donor splenocyte transfusion (5 × 10^6^ H-2^d^ cells, i.v.) at the time of engraftment (31, 32). All transplant studies involved 5 allografts/group and were repeated at least once, with similar results.

### In Vivo Studies of Treg Cells.

Treg cell survival was assessed by adoptive transfer of 0.5 × 10^6^ *mtDNA^B6^* or *mtDNA^NZB^* CD4^+^Foxp3^+^ Treg cells plus 0.1 × 10^6^ *mtDNA^B6^* Tconv CD4^+^ cells (4 mice/group) and assayed by flow cytometry, the proportions and absolute numbers of Tregs within lymph nodes and spleens assessed 30 d later (50). In homeostatic proliferation assays, congenic Thy1.1 + CD4^+^CD25^−^ *mtDNA^B6^* cells purified on MACS columns were mixed in a 1:1 ratio with Thy1.2 + CD4^+^CD25^+^ T cells from *mtDNA^B6^* or *mtDNA^NZB^* donors and adoptively transferred into *Rag1^−/−^* mice. After 7 d, recipient spleens and lymph nodes were collected and the total number of Thy1.1 + CD4^+^ T cells was determined by flow cytometry (50).

### T Cell Activation.

Isolated T cells from the T conv CD4^+^ *mtDNA^B6^* versus *mtDNA^NZB^* mice were divided into two groups for each cell type: resting and activated. To activate the cells, CD28/CD3ε beads (Miltenyi) plus IL-2 were added to Treg cells (2:1, beads: cells) for 2 h and to Tconv CD4^+^ cells (1:1, beads: cells) for 72 h.

### Library Preparation and Sequencing.

RNA was isolated from the cells using the RNeasy Micro kit standard protocols (QIAGEN). The amount and quality of the RNA were tested by Qubit and TapeStation. RNA-seq libraries were prepared at the Crown Genomics Institute of the Nancy and Stephen Grand Israel National Center for Personalized Medicine, Weizmann Institute of Science. A bulk adaptation of the MARS-Seq protocol (51, 52) was used to generate RNA-Seq libraries for expression profiling. Briefly, 60 ng of input RNA from each sample was barcoded during reverse transcription and then pooled. Following Agencourt AMPure XP beads cleanup (Beckman Coulter), the pooled samples underwent second strand synthesis and were linearly amplified by T7 in vitro transcription. The resulting RNA was fragmented and converted into a sequencing-ready library by tagging the samples with Illumina sequences during ligation, reverse transcription (RT), and PCR. Libraries were quantified with Qubit and TapeStation as well as by qPCR for the ActB gene as previously described (51, 52). Sequencing was carried out on NextSeq 500 (Illumina Inc.) using the high output kit, producing 400 M, 75 bp single end reads.

### Bioinformatics Analysis.

The RNA sequence read data were preprocessed including the trimming of Poly-A/T stretches and Illumina adapters using cutadapt (53). Resulting reads shorter than 30 bp were discarded. The reads were then mapped to 3′ UTR regions (1,000 bases) of the *Mus musculus* GRCm39 genome according to Ensembl annotations (54) version 106, using STAR (55) with EndToEnd option and with the outFilterMismatchNoverLmax was set to 0.05. Deduplication was carried out by flagging all reads that were mapped to the same gene and had the same unique molecule identifier (UMI). Counts for each gene were quantified using htseq-count (56) based on the annotations mentioned above. UMI counts were corrected for saturation by considering the expected number of unique elements when sampling without replacement. Differentially expressed genes were identified using DESeq2 (57) with the betaPrior, cooksCutoff, and independentFiltering parameters set to False. Raw *P* values were adjusted for multiple testing using the procedure of Benjamini and Hochberg (58). The pipeline was run using snakemake (59). Volcano plots were generated using the DESeq2 (57) data and the EnhancedVolcano R package with an adjusted p-value cutoff of ≤0.05. Enriched GO Terms and KEGG pathways were identified from the DESeq2 data via the ClusterProfiler (60) R package using the pipeline in https://github.com/jahaltom/MITOCHONDRIAL-TUMOR/tree/main. An adjusted *P*-value cutoff of ≤0.05 was used. Heatmaps were generated using the pheatmap R package.

### ROS Detection by Flow Cytometry.

ROS production in resting and activated (CD4/CD28 beads, 2 h) of *mtDNA^B6^*, *mtDNA^NZB^*, or *mtDNA^NZB^mCAT*^fl^ (Vav^iCre^) Treg and Tconv CD4^+^ cells were quantified using a CellRox Green Flow Cytometry Kit (Thermo Fisher Scientific) (28).

### Mitochondrial Respiration and ROS Production.

Mitochondrial respiration and mROS production were measured using High-resolution FluoRespirometry (Oroboros Instruments) with FluoSensor green at excitation (525 nm) and detection of Amplex Red fluorescence. An air calibration was performed with MiR05 buffer supplemented with 15 µM DTPA (diethylenetriaminepentaacetic acid). Subsequently, Amplex Red (10 µM), horseradish peroxidase (HRP) (1 U/mL), and SOD (2 U/mL) were added, and the fluorescence signal was calibrated by two consecutive additions of H_2_O_2_ (0.2 µM each). Additional ROS calibrations were performed after addition of the sample and at the end of the titration protocol. Mice were killed using cervical dislocation, and the bone marrows were dissected and homogenized in MiR05+DTPA using Dounce homogenizers. The homogenized bone marrows were added to the respirometry chambers. A substrate-uncoupler-inhibitor titration (SUIT-) protocol was used to evaluate mitochondrial respiration and ROS production at different mitochondrial respiratory states. Titration protocol: pyruvate, malate, and glutamate (5 mM, 0.5 mM, and 10 mM), ADP (5 mM), succinate (10 mM), oligomycin (2.5 µM), FCCP (1.5 µM), rotenone (0.5 µM), and Antimycin A (2.5 µM). After each addition, a plateau in respiration and ROS production was awaited. Respirometry chambers were reoxygenated by opening for 5 min after Oligomycin and after antimycin A. Respiration was corrected for the residual oxygen consumption after Antimycin A. ROS production was first corrected for the background flux before the addition of any sample. Subsequently, it was corrected for the O_2_ concentration at each respective interval according to an O_2_-ROS correlation curve obtained by reoxygenating after Antimycin A. The absolute ROS levels of the *mCAT^fl^ Vav^iCre^* mice were normalized to the ROS levels of the *mCAT^fl^* mice.

### Associations.

DCW is a consultant for Pano Pharmaceuticals and Medical Excellence Capital.

## Supplementary Material

Appendix 01 (PDF)

Dataset S01 (XLSX)

Dataset S02 (PDF)

## Data Availability

Research materials are available from WallaceD1@chop.edu. Bulk RNA-Seq data can be found at the Gene Expression Omnibus under accession Nos. GSE276714 (33) and GSE280896 (34).
