# Supplementary material for: Mitochondrial DNA lineages determine tumor progression through T cell reactive oxygen signaling
Source: Proc Natl Acad Sci U S A. 2025 Jan 3;122(1):e2417252121. doi: 10.1073/pnas.2417252121 (PMC11725793; doi:10.1073/pnas.2417252121)
Supplement: Supplementary file 1 — Appendix 01 (PDF) [file pnas.2417252121.sapp.pdf]

PNAS

Supporting Information for
MITOCHONDRIAL DNA LINEAGES DETERMINE TUMOR PROGRESSION THROUGH T CELL REACTIVE OXYGEN SIGNALING

Tal Yardeni[1,2], Arnold Z Olali[1], Hsiao-Wen Chen[1], Liqing Wang[3], Jeff Halton[1], Angi Zenab[2], Ryan Morrow[1], Arrienne Butic[1], Deborah G Murdock[1,4], Katrina G. Waymire[5], Grant R. MacGregor[5], Ben Boursi[6], Ulf H. Beier[7], Wayne W. Hancock[1,3], and Douglas C. Wallace[1,4,8]

Corresponding author: Douglas C. Wallace.

Email: wallaced1@chop.edu

This PDF file includes:

Supporting text
Figures S1 to S7

Other supporting materials for this manuscript include the following:

Datasets S1
Datasets S2
Datasets S3

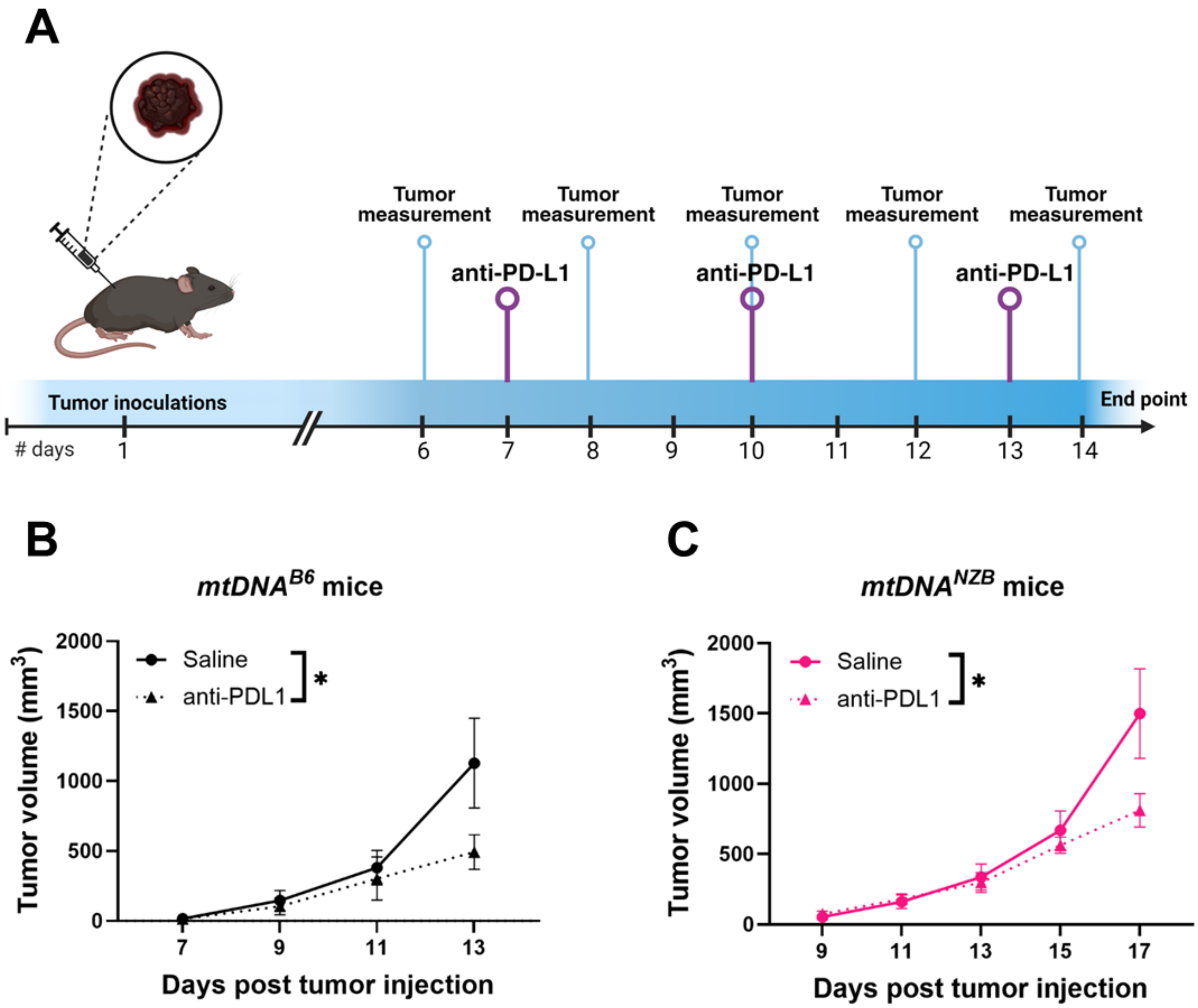

Fig. S1. Differential *mtDNA^{B6}* and *mtDNA^{NZB}* rejection of melanoma cells did not significantly respond to anti-PD-L1 immune checkpoint therapy. (**A**) Experiment protocol. (**B-C**) B16F10 melanoma cells (1X10^5) were injected into the flank of (**B**) *mtDNA^{B6}* mice and (**C**) *mtDNA^{NZB}* mice, followed at days 7,10,13 and 16 with an injection of 100 µg of anti-PD-L1 and tumor growth monitored on the days indicated in the figures, N =12-16/group, repeated twice, two-way ANOVA, * $p<0.05$.

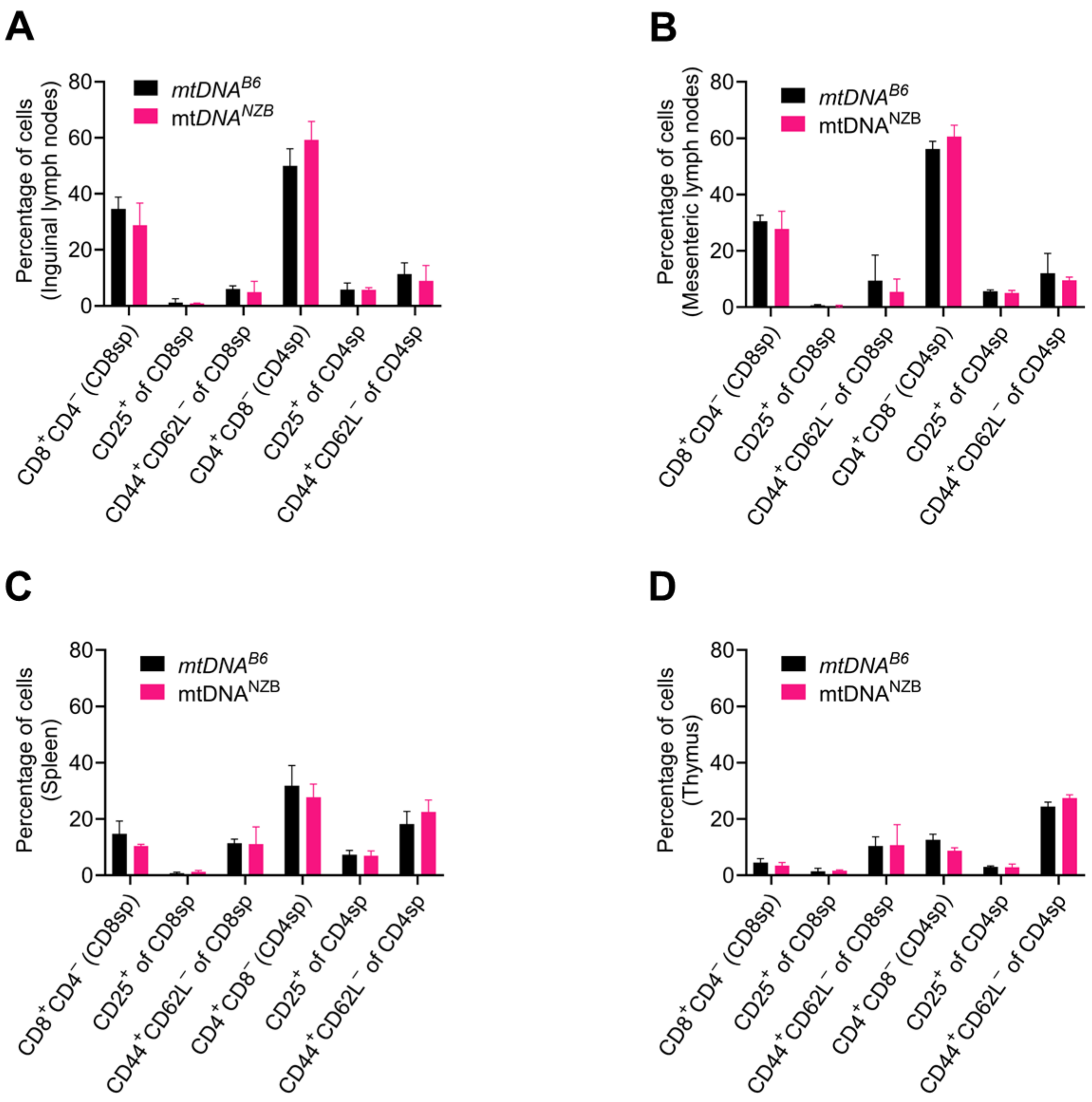

Fig. S2. ***mtDNA*^{B6} and *mtDNA*^{NZB} haplotypes exhibit no difference in resting immune cell populations**. T cells were isolated and counted from (**A**) inguinal lymph nodes, (**B**) mesenteric lymph nodes, (**C**) spleen, and (**D**) thymus. *mtDNA*^{B6} (black) and *mtDNA*^{NZB} (pink) cells, N = 3/group, Mann-Whitney test. Mean ± SEM. Abbreviations: sp, single positive.

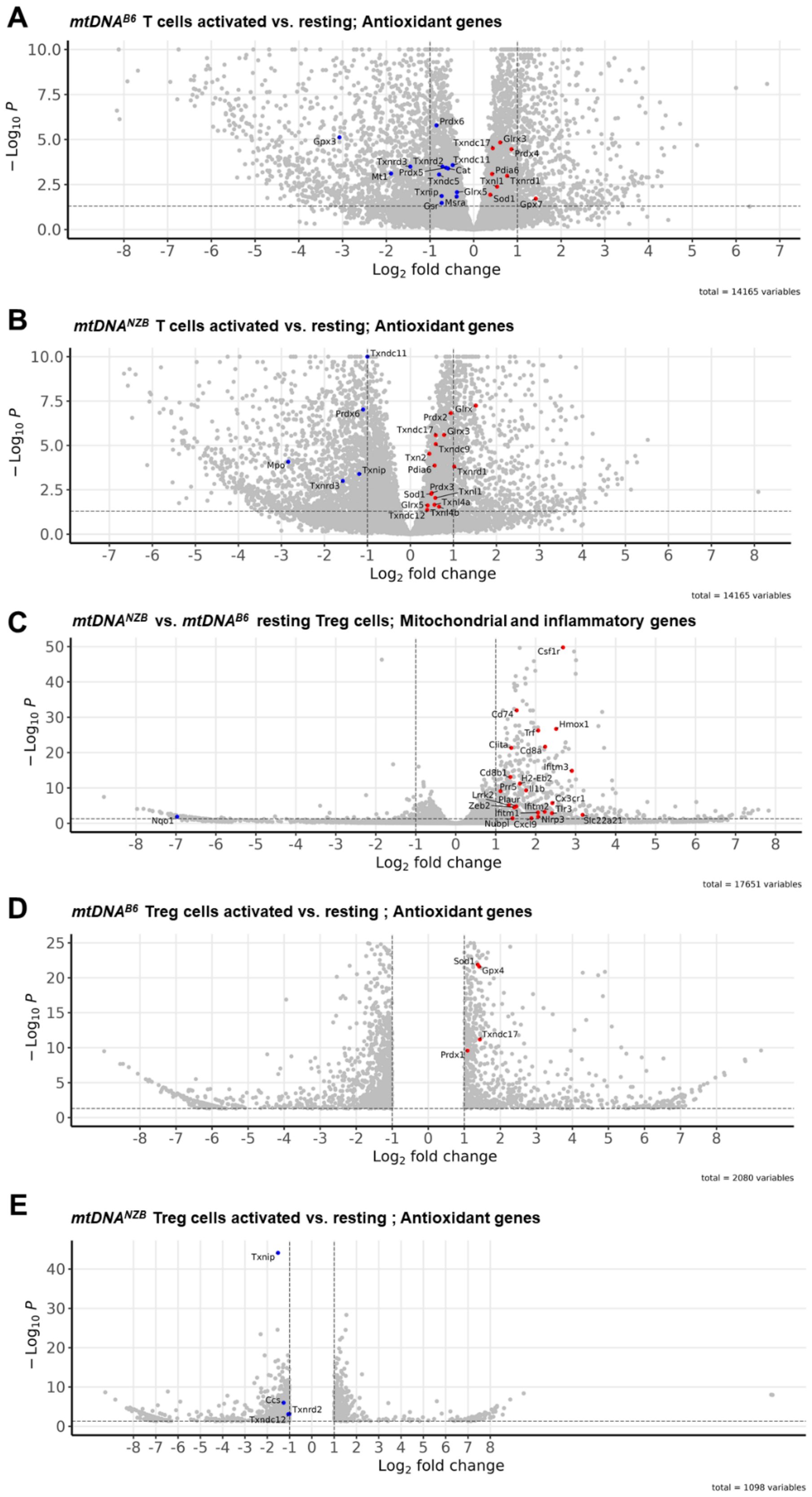
A
mtDNA^B6 T cells activated vs. resting; Antioxidant genes
Prdx6
Gpx3
Txndc17
Glrx3
Prdx4
Txnrd3
Txnrd2
Txndc11
Prdx5
Cat
Pdia6
Mt1
Txndc5
Txnl1
Txnrd1
Txnip
Glrx5
Sod1
Gsr
Msra
Gpx7
− Log10 P
Log2 fold change
total = 14165 variables
B
mtDNA^NZB T cells activated vs. resting; Antioxidant genes
Txndc11
Prdx6
Glrx
Prdx2
Txndc17
Glrx3
Txndc9
Txn2
Mpo
Txnip
Pdia6
Txnrd1
Txnrd3
Prdx3
Sod1
Txnl1
Glrx5
Txnl4a
Txndc12
Txnl4b
− Log10 P
Log2 fold change
total = 14165 variables
C
mtDNA^NZB vs. mtDNA^B6 resting Treg cells; Mitochondrial and inflammatory genes
Csf1r
Cd74
Hmox1
Trf
Ciita
Cd8a
Ifitm3
Cd8b1
H2-Eb2
Prf5
Il1b
Lrrk2
Plaur
Cx3cr1
Zeb2
Ifitm2
Tlr3
Ifitm1
Nlrp3
Nqo1
Nubpl
Cxcl9
Slc22a21
− Log10 P
Log2 fold change
total = 17651 variables
D
mtDNA^B6 Treg cells activated vs. resting ; Antioxidant genes
Sod1
Gpx4
Txndc17
Prdx1
− Log10 P
Log2 fold change
total = 2080 variables
E
mtDNA^NZB Treg cells activated vs. resting ; Antioxidant genes
Txnip
Ccs
Txnrd2
Txndc12
− Log10 P
Log2 fold change
total = 1098 variables

Fig. S3. *mtDNA^{B6}* and *mtDNA^{NZB}* haplotypes modulate gene expression in activated Tconv and Treg CD4^{+} cell, with antioxidant gene expression changes highlighted. (A-B) Volcano plot of the genes significantly up- versus downregulated in activated **(A)** *mtDNA^{B6}* and (**B**) *mtDNA^{NZB}* Tconv CD4^{+} cells, with all antioxidant genes labeled**.** (**C**) Volcano plot of the genes significantly up- versus downregulated in *mtDNA^{B6}* vs. *mtDNA^{NZB}* Treg cells, with all mitochondrial and inflammatory genes labeled. **(D-E)** Volcano plot of the genes significantly up- versus downregulated in activated (**D**) *mtDNA^{B6}* and (**E**) *mtDNA^{NZB}* Treg cells, with all antioxidant genes labeled**.** (n=3-5/ model). Red = upregulated, blue= downregulated.

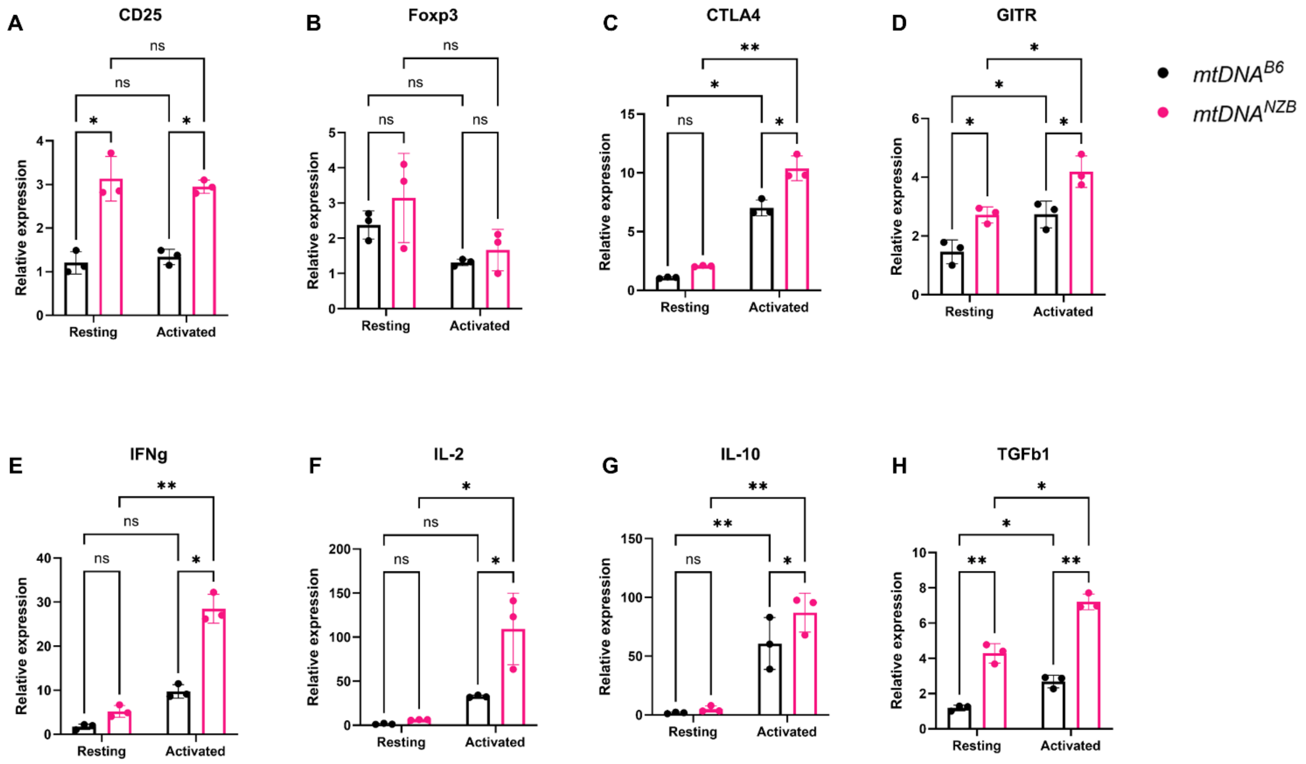

Fig. S4. Reverse transcriptase-quantitative polymerase chain reaction (RT-qPCR) analysis of CD4^{+} Tregs mRNA levels. Tregs were isolated from *mtDNA*^{B6} (black) and *mtDNA*^{NZB} (pink) mice and cultured under resting conditions or activated for 2 hrs and their mRNA levels quantified by RT-qPCR.   One-way ANOVA, ns, not significant, *p<0.05 and **p<0.01.

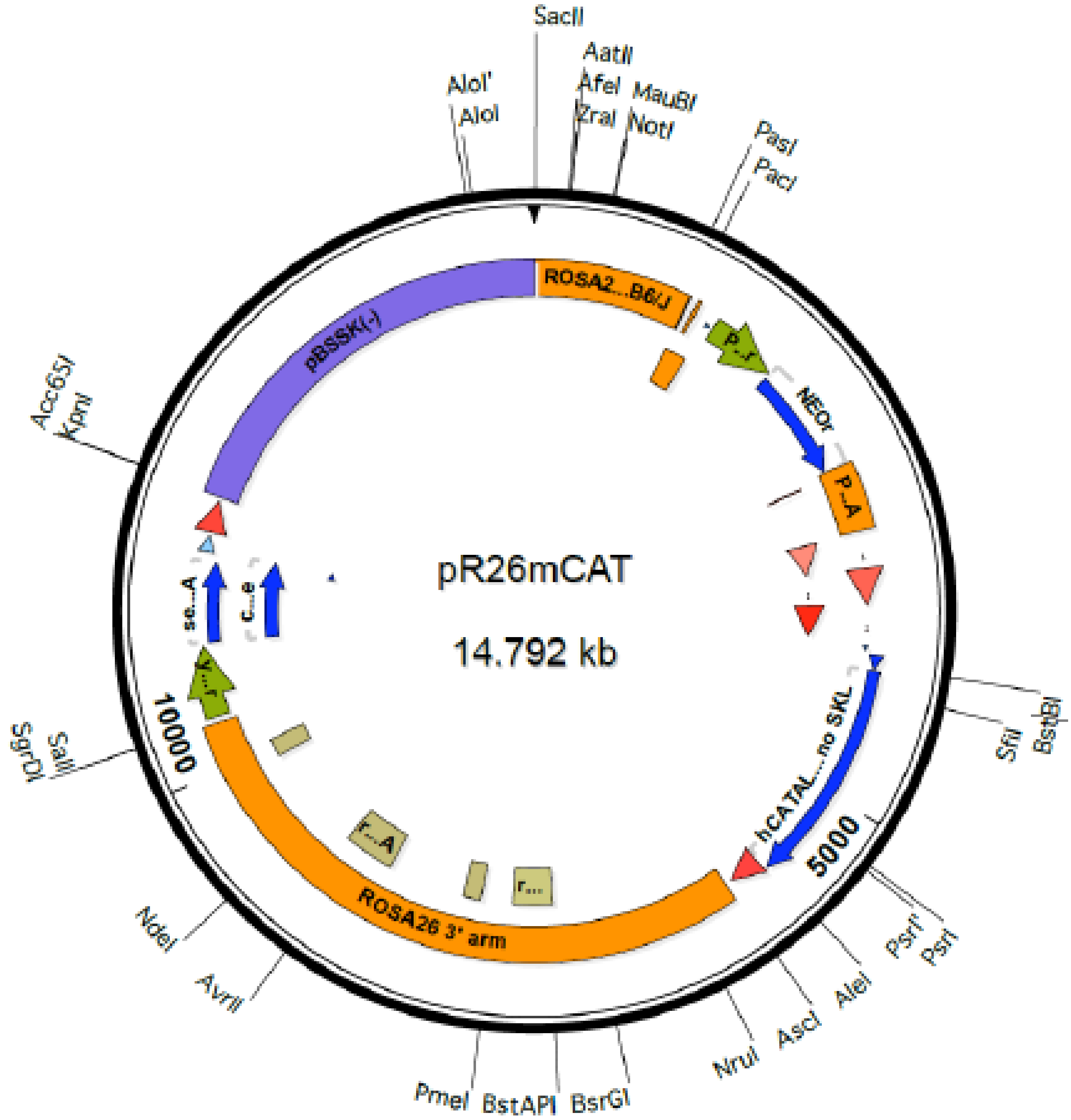

Fig. S5. **Structure of the *Rosa26-Stop^{fl}-mCAT* plasmid.** Circular map of the Rosa26-LSL-mCAT targeting plasmid with the inserted elements described under “Mice” in **Materials and Methods.**

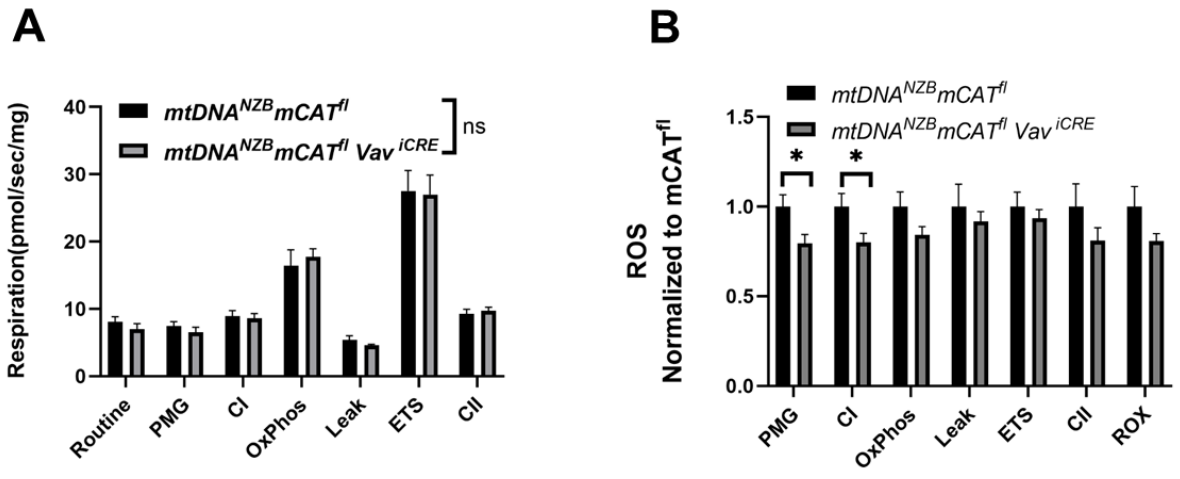

Fig. S6. **mCAT expression in the bone marrow decreases reactive oxygen species without affecting respiration.** Mitochondrial (**A**) respiration and (**B**) ROS production measured by the high-resolution respirometry, Oroboros O2k. Detailed respiration and ROS measurements made during routine, basal respiration; PMG, complex I-linked leak respiration; ADP, ADP stimulated; Suc, OXPHOS capacity with succinate; Olg, leak respiration with Oligomycin; FCCP, uncoupled mitochondria; Rot, rotenone inhibition of complex I inhibition demonstrating complex II-linked respiration; AA, antimycin A complex III inhibition demonstrating residual oxygen consumption. n = 5 per group, Mann-Whitney test for each state, *P < 0.05.

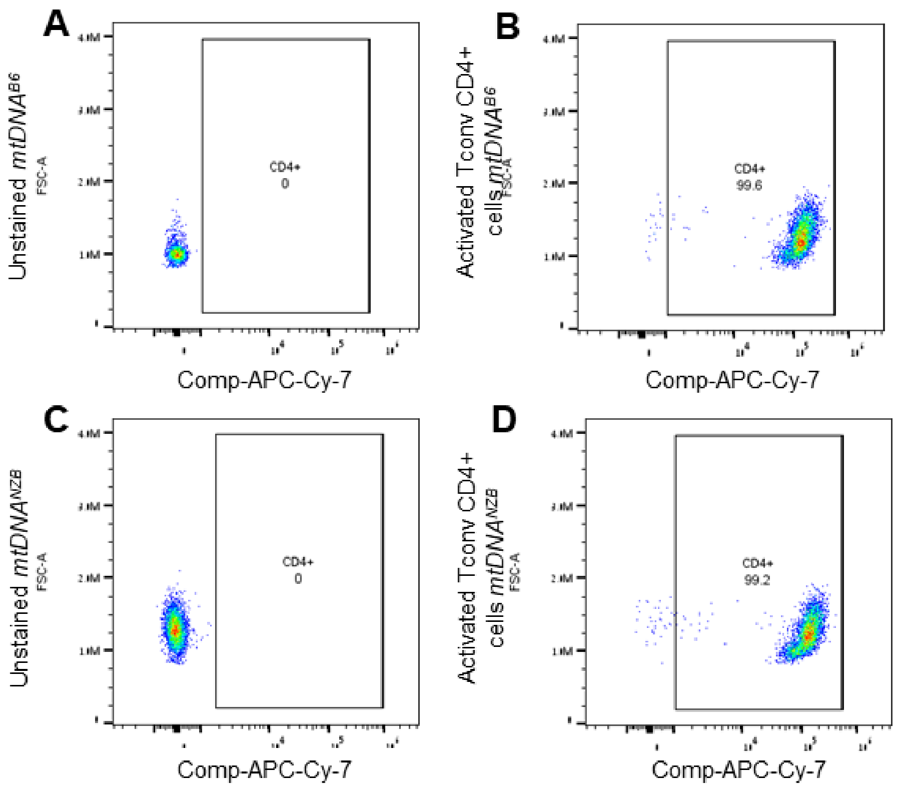

Fig. S7. **Gating analysis for isolated CD4$^+$ cells from *mtDNA^{B6}* and *mtDNA^{NZB}* mice.** Flow cytometry analysis of Tconv CD4$^+$ cells was performed to assess CD4$^+$ expression in activated and resting conditions. APC-Cy7 was used to label the CD4$^+$ population. The sequential gating strategy ensured accurate identification of CD4$^+$ cells. (**A-B**) CD4$^+$ expression in *mtDNA^{B6}* Tconv cells. (**A**) Unstained, (**B**) CD4+ staining for activated Tconv CD4+ cells. (**D-E**) CD4$^+$ expression in *mtDNA^{NZB}* Tconv cells. (**D**) unstained, (**E**) CD4+ staining for activated Tconv CD4+ cells.

Dataset S1: Yardeni T, Olali AZ, Chen HW, Wang L, Halton J, Zenab A, Morrow R, Butic A, Murdock DG, Waymire KG, MacGregor GR, Boursi B, Beier UH, Hancock WH, and Wallace DC. Data from " Mitochondrial DNA lineages determine tumor progression through T cell reactive oxygen signaling". GSE276714. Available at www.ncbi.nlm.nih.gov/geo/query/acc.cgi?acc=GSE276714. Deposited 5 September 2024.

Dataset S2: Yardeni T, Olali AZ, Chen HW, Wang L, Halton J, Zenab A, Morrow R, Butic A, Murdock DG, Waymire KG, MacGregor GR, Boursi B, Beier UH, Hancock WH, and Wallace DC. Data from " Mitochondrial DNA lineages determine tumor progression through T cell reactive oxygen signaling". GSE280896. Available at www.ncbi.nlm.nih.gov/geo/query/acc.cgi?acc=GSE280896. Deposited 1 November 2024.

Dataset S3 (separate file). Structure of the *Rosa26-Stop^{fl}-mCAT* plasmid. The complete annotated sequence of the Rosa26-LSL-mCAT construct.
