# Supplementary figures and images for: Mitochondrial DNA lineages determine tumor progression through T cell reactive oxygen signaling
Source: Proc Natl Acad Sci U S A. 2025 Jan 3;122(1):e2417252121. doi: 10.1073/pnas.2417252121 (PMC11725793; doi:10.1073/pnas.2417252121)

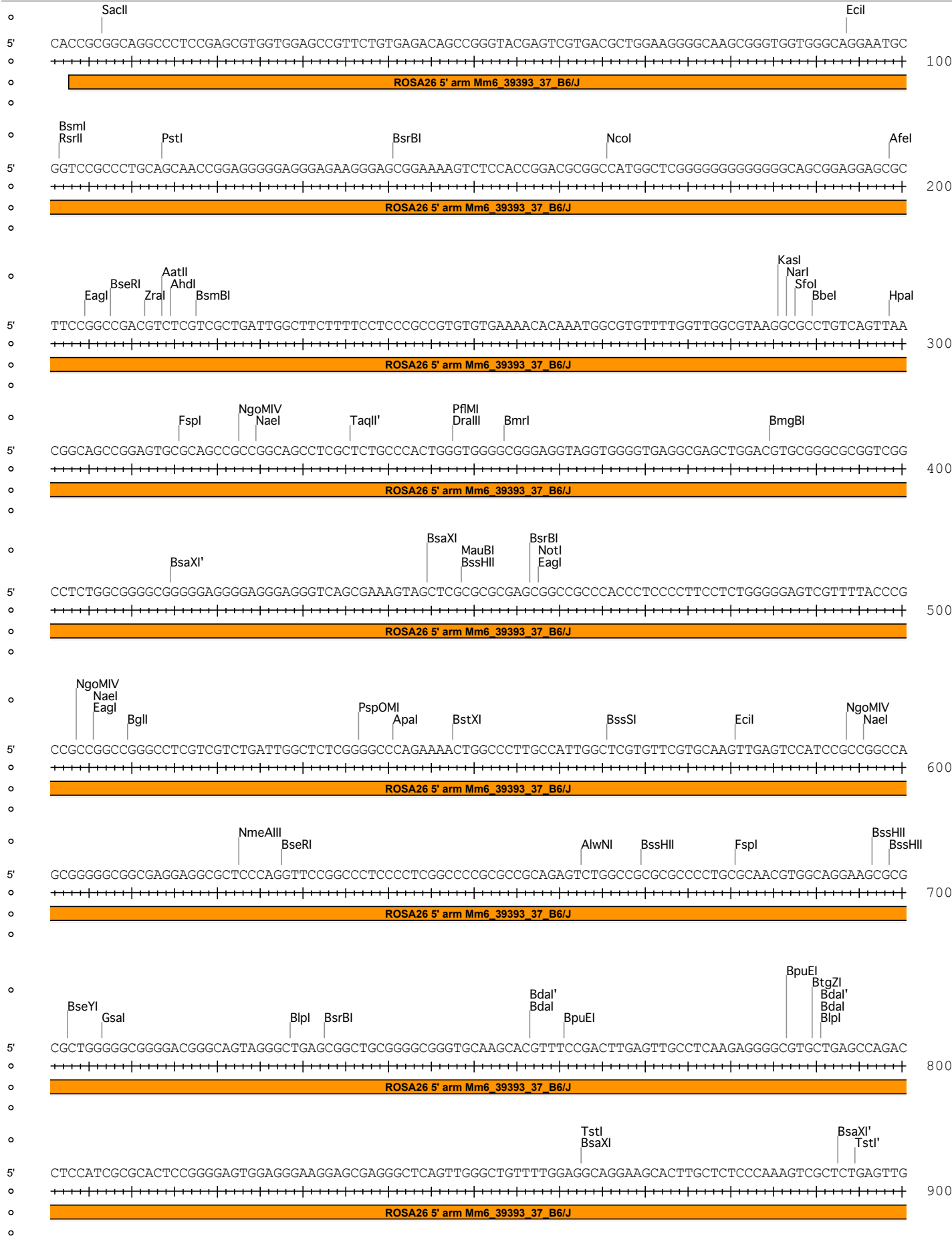

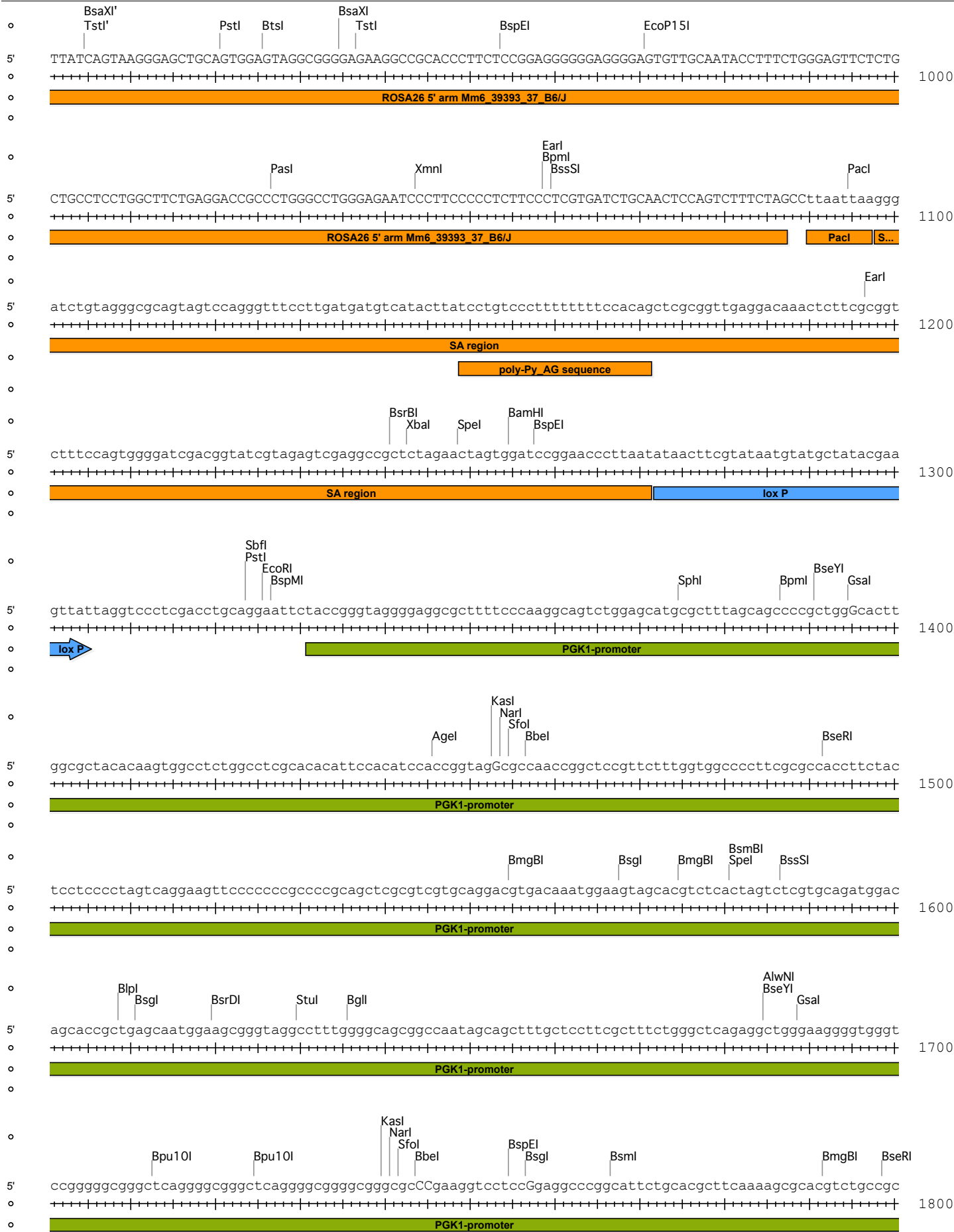

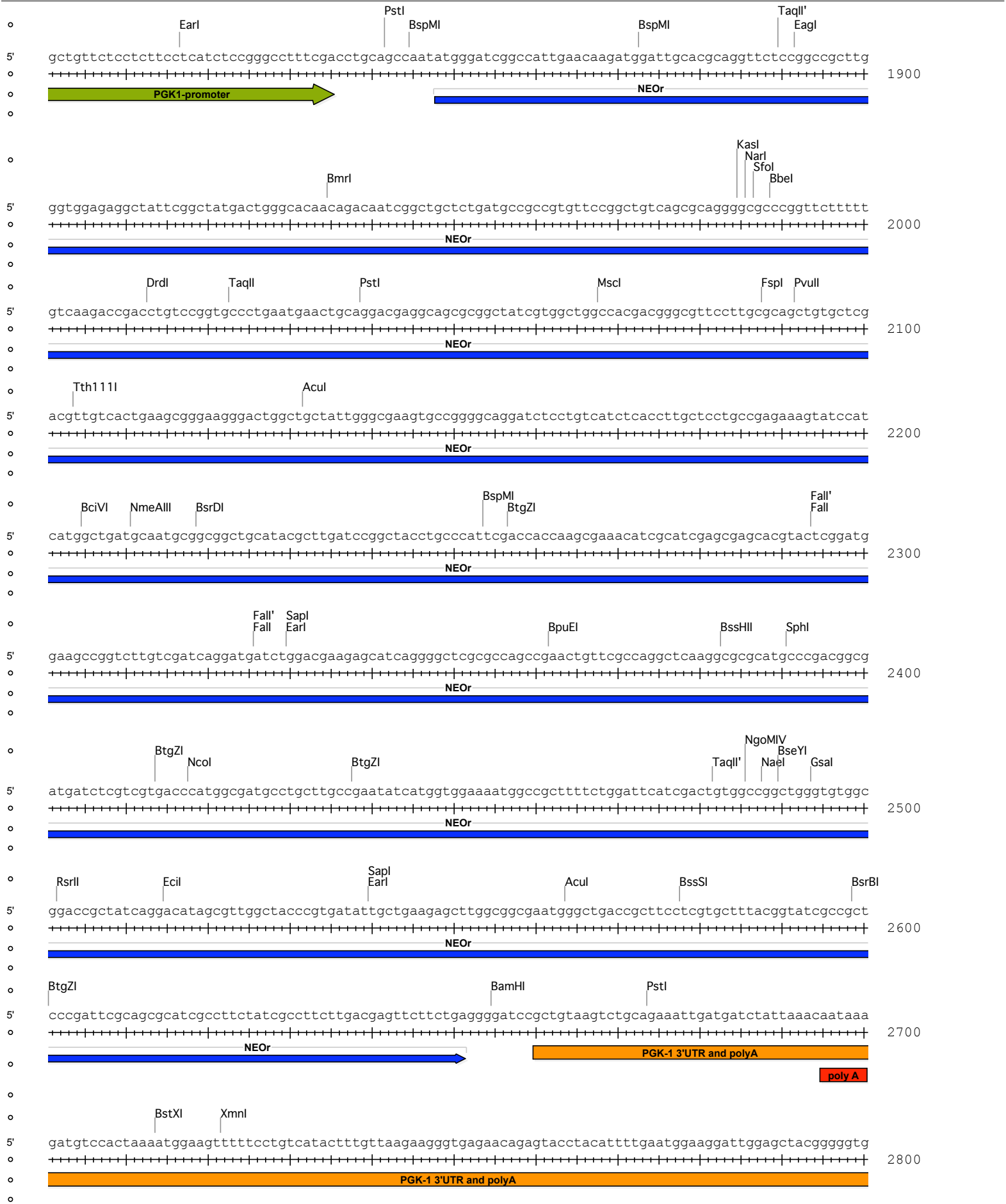

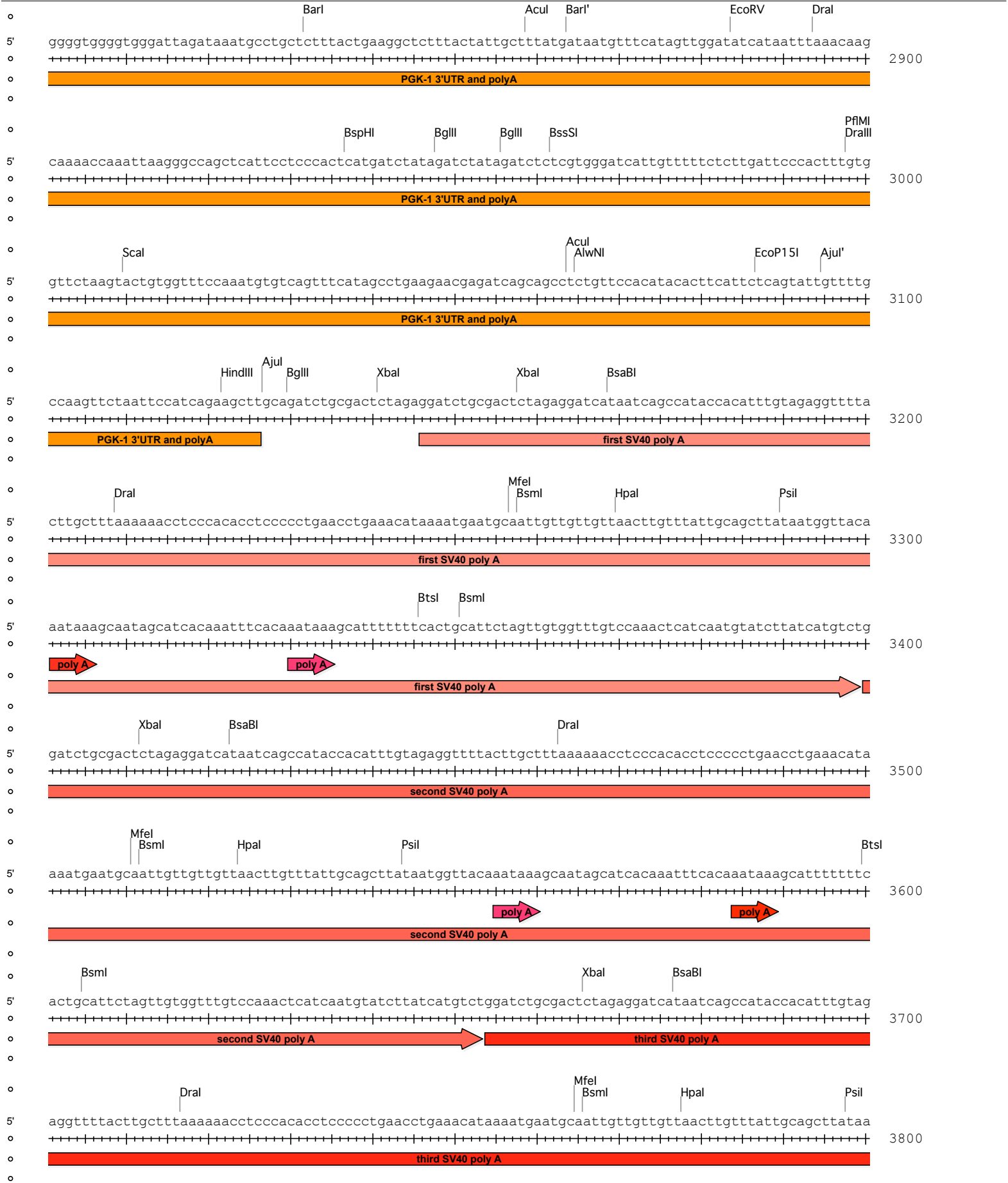

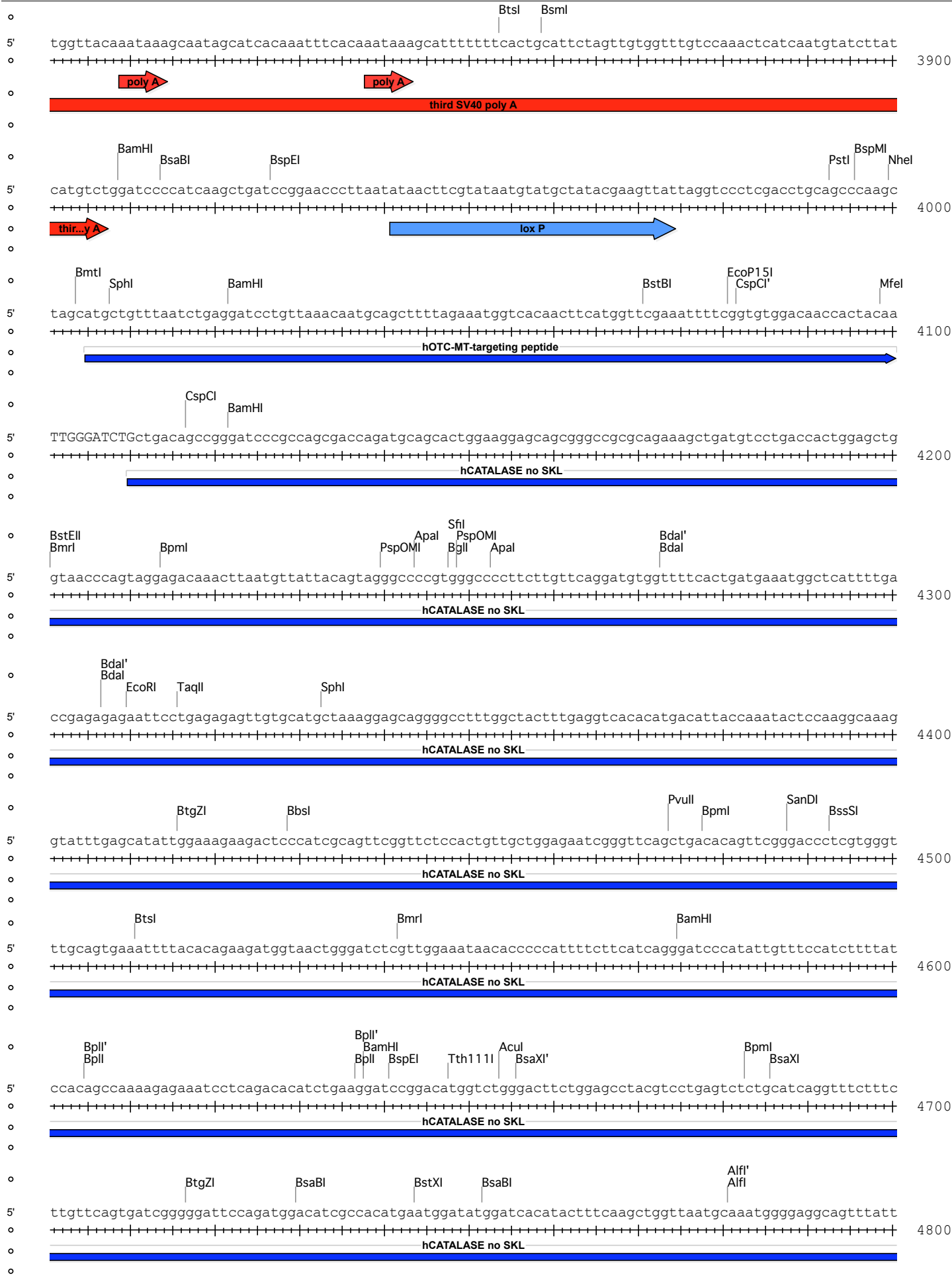

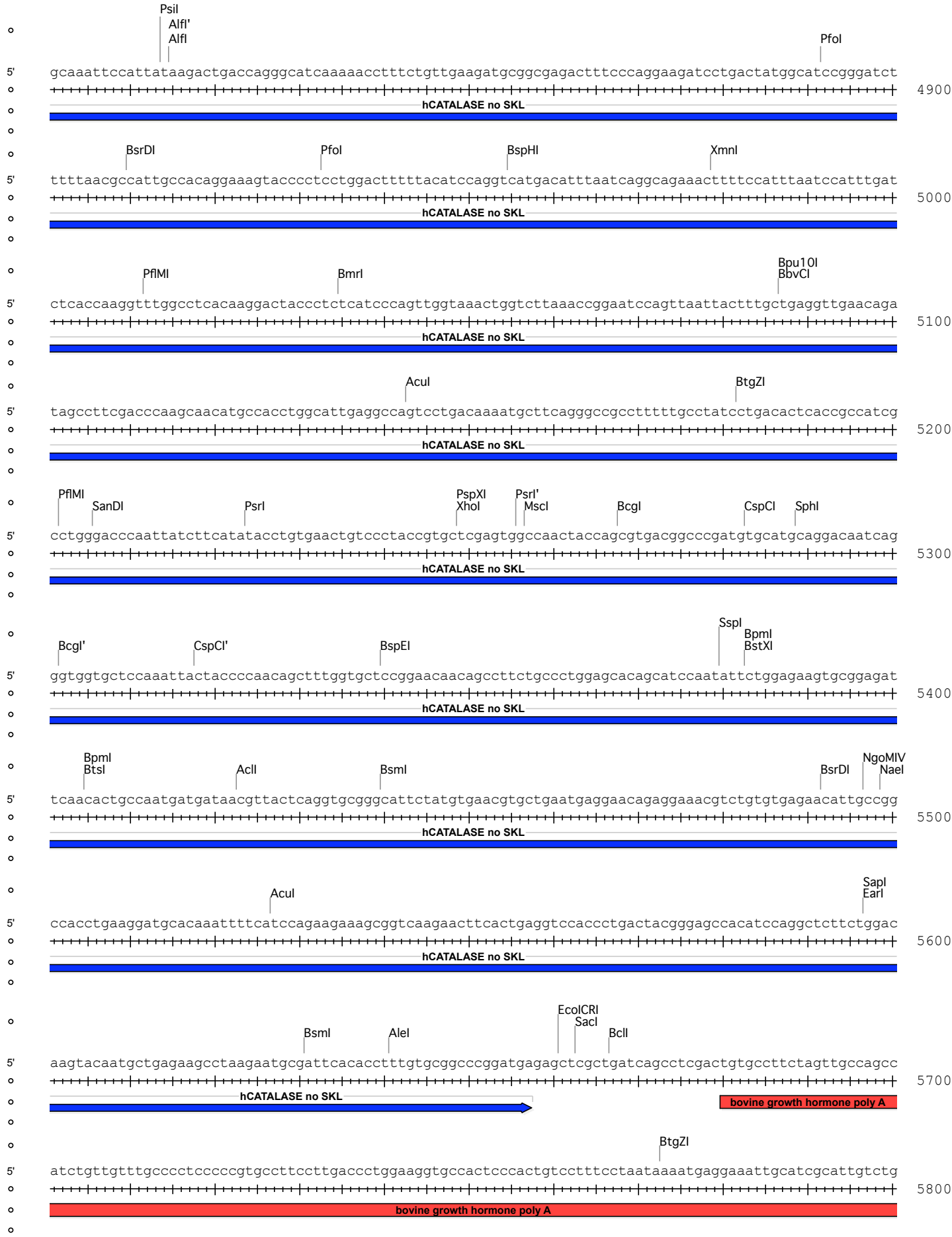

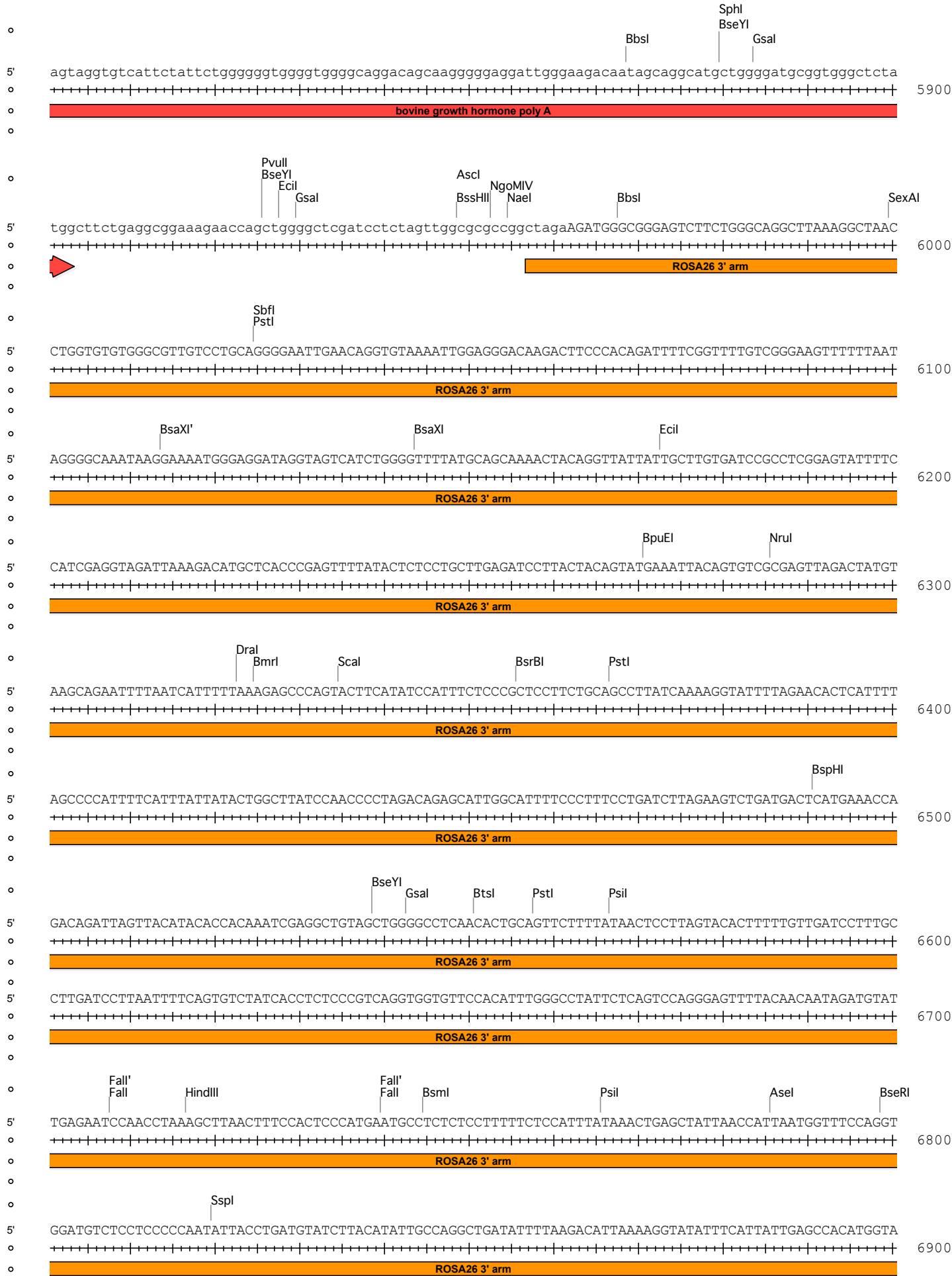

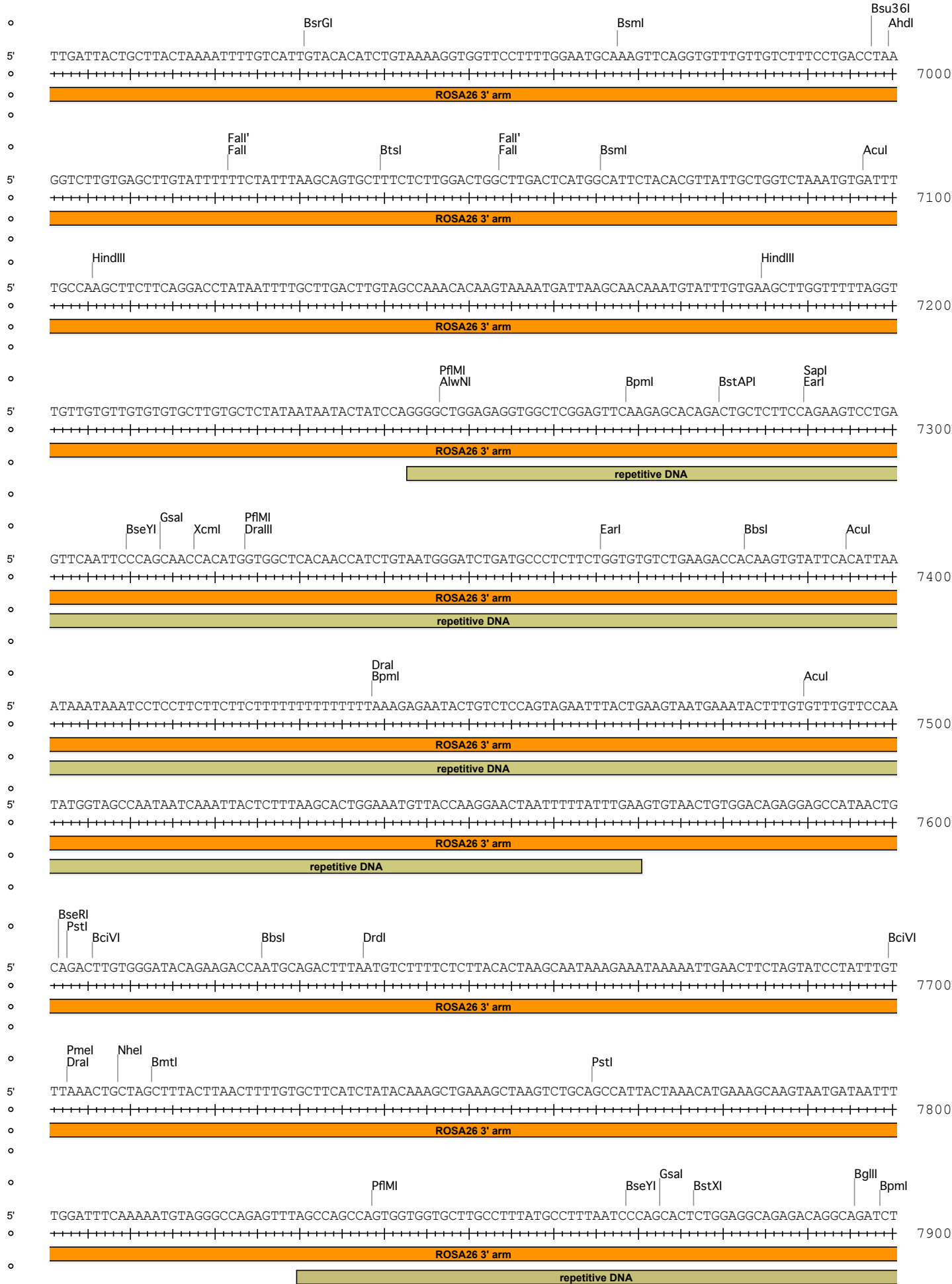

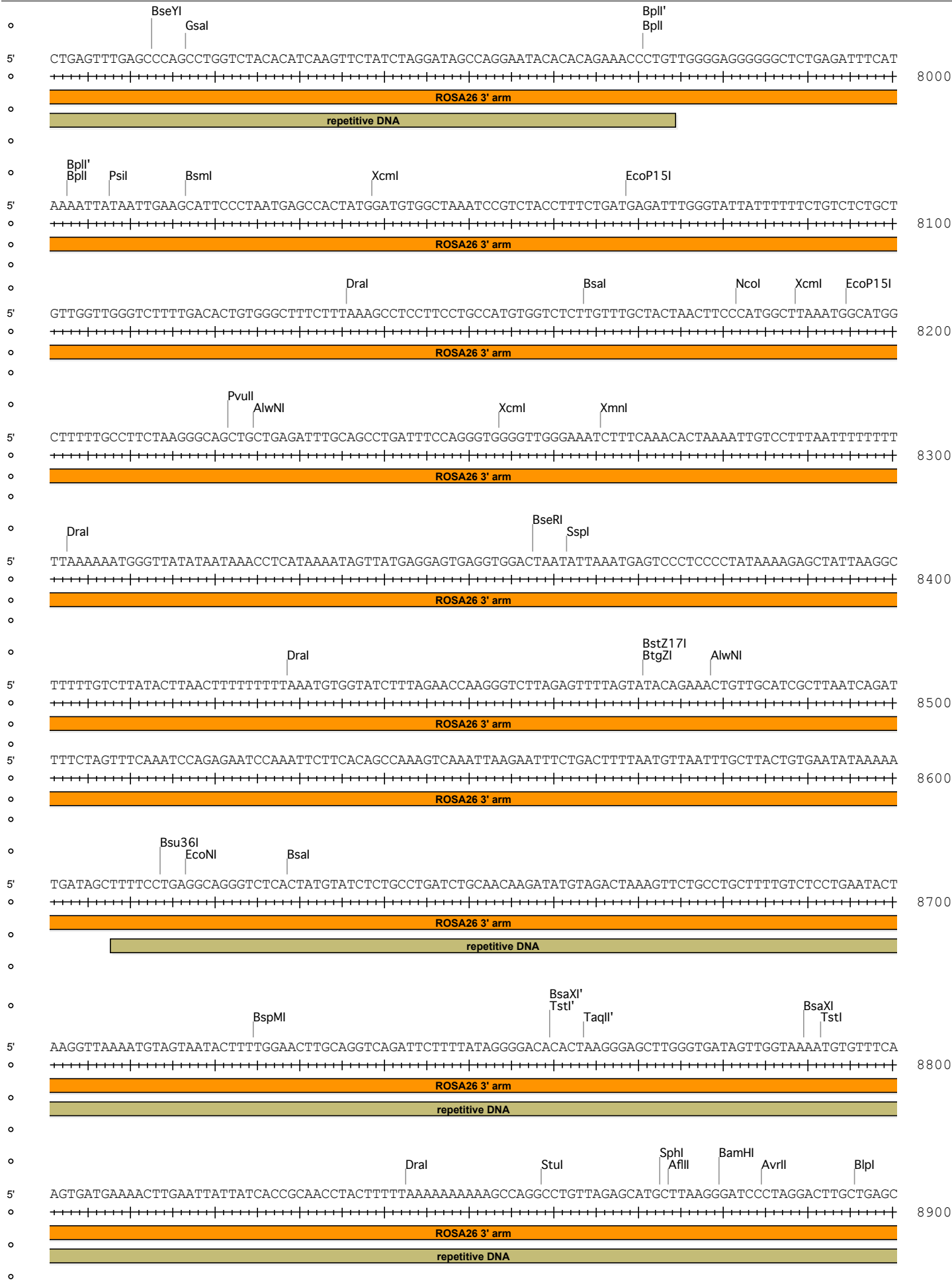

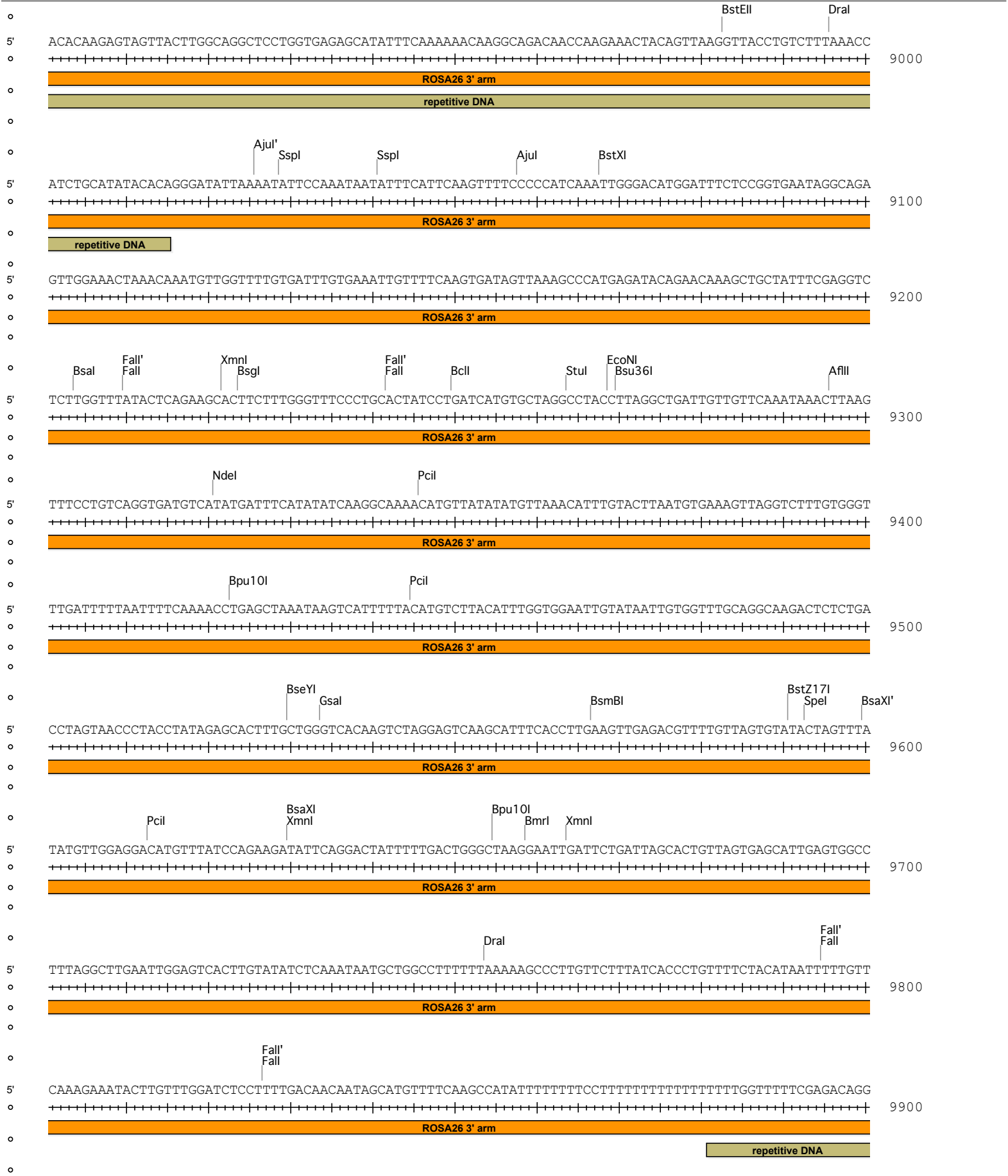

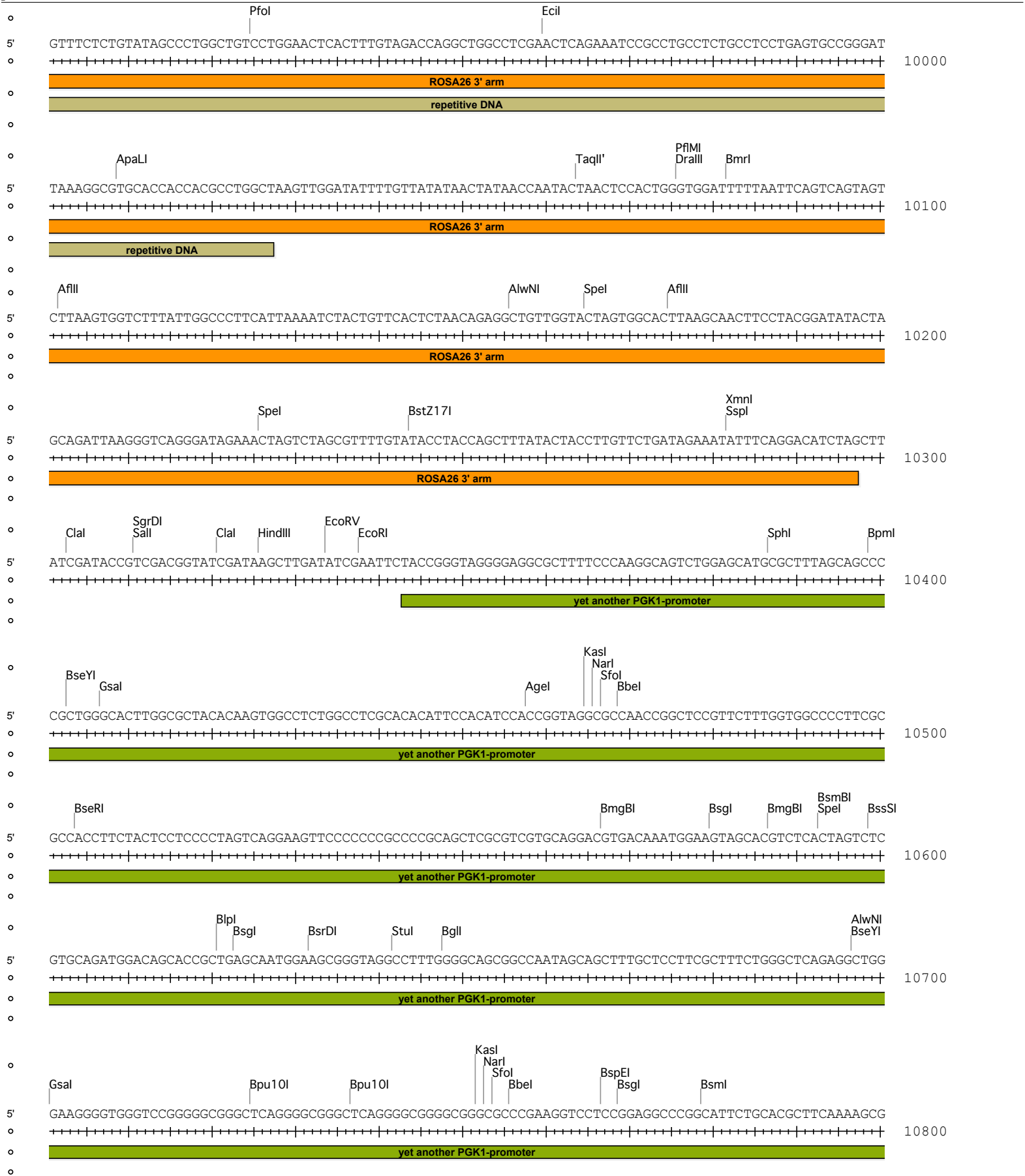

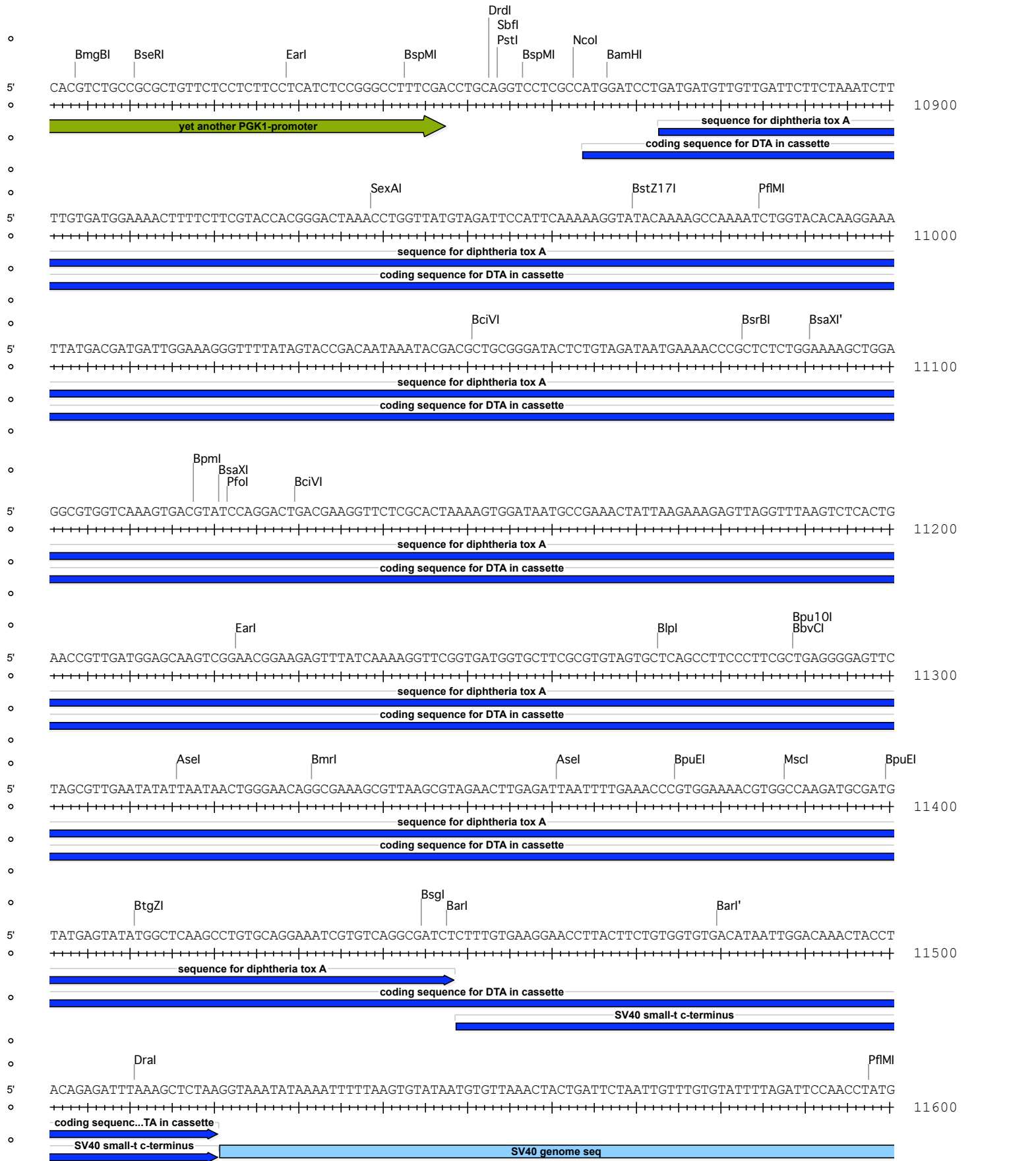

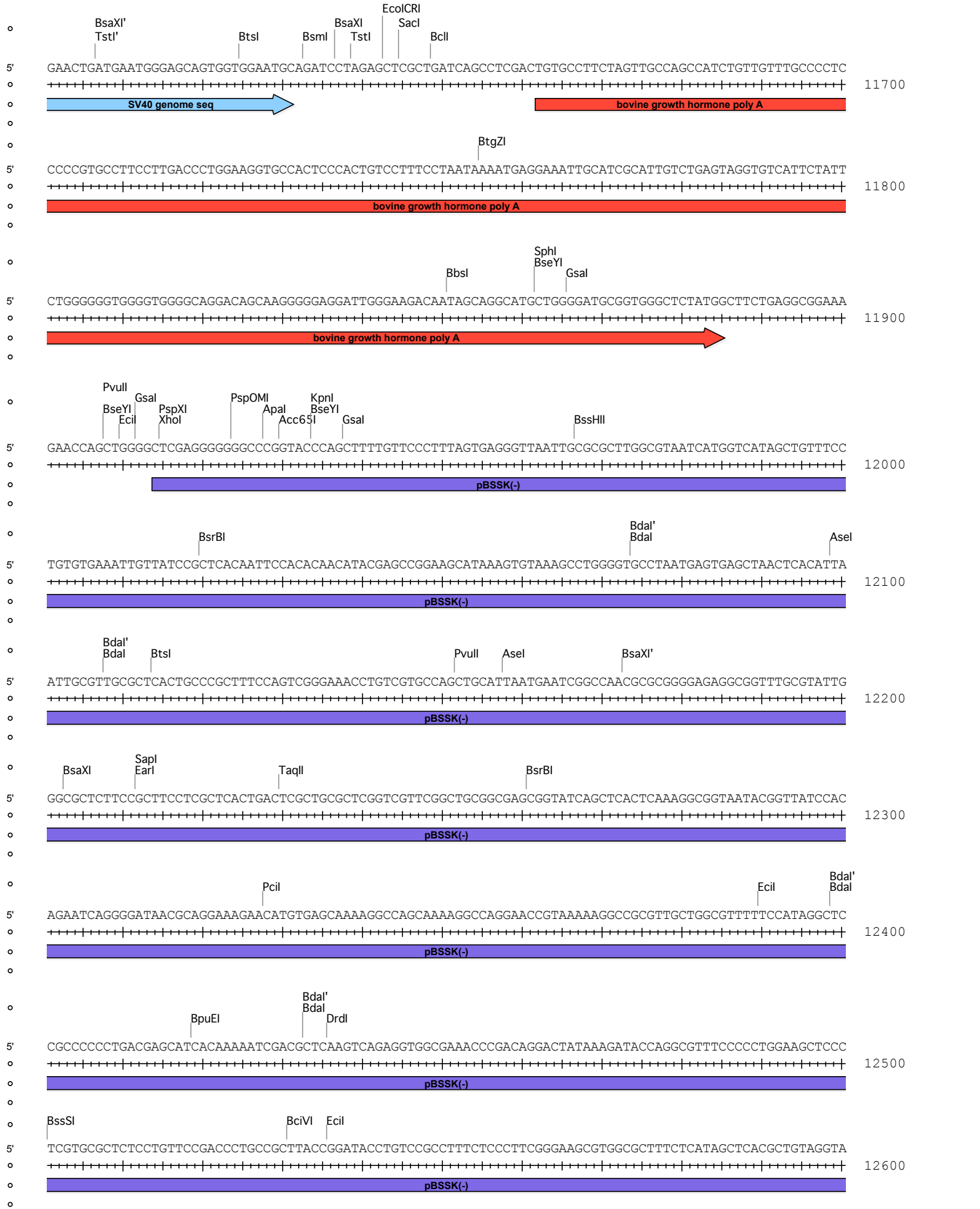

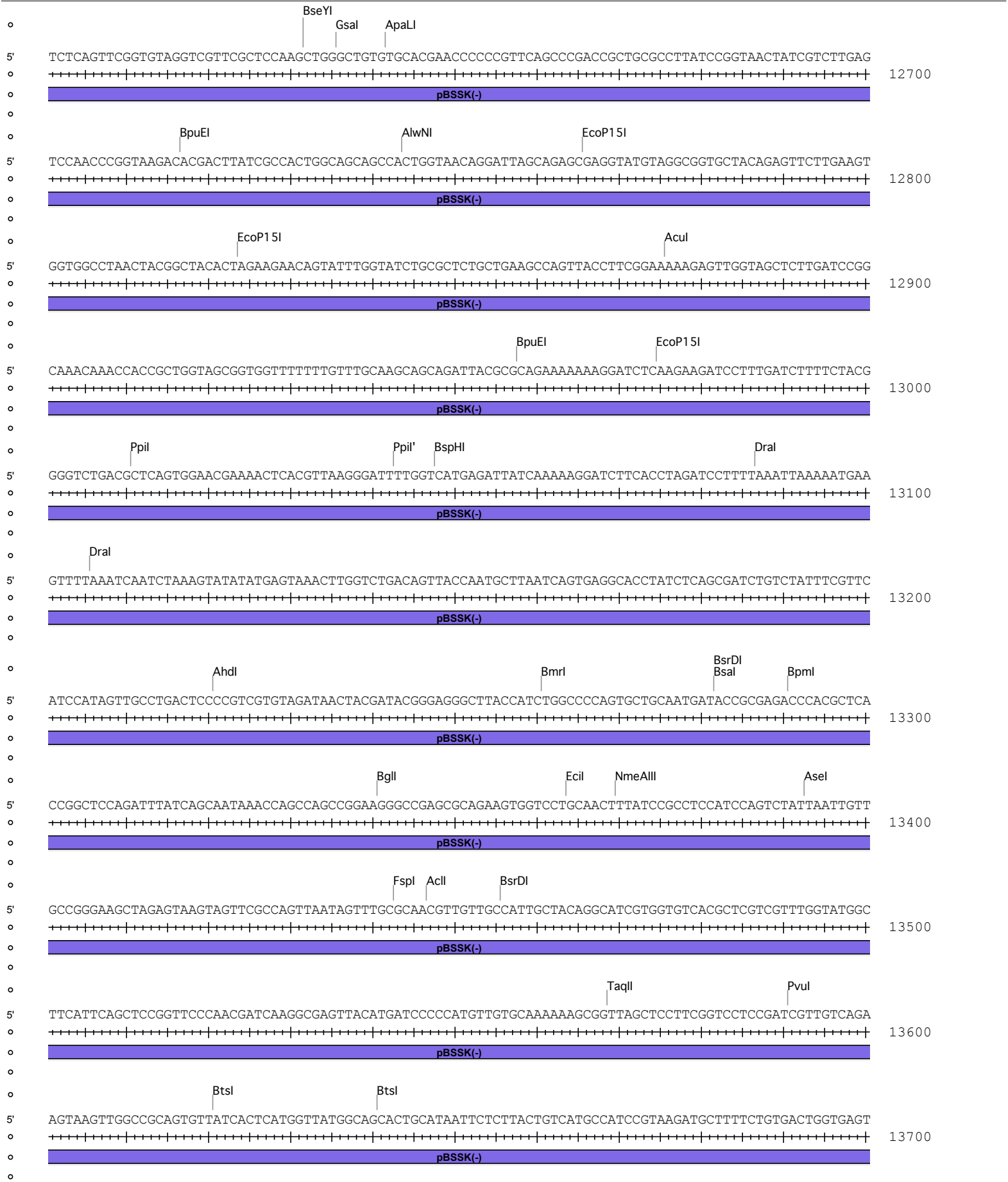

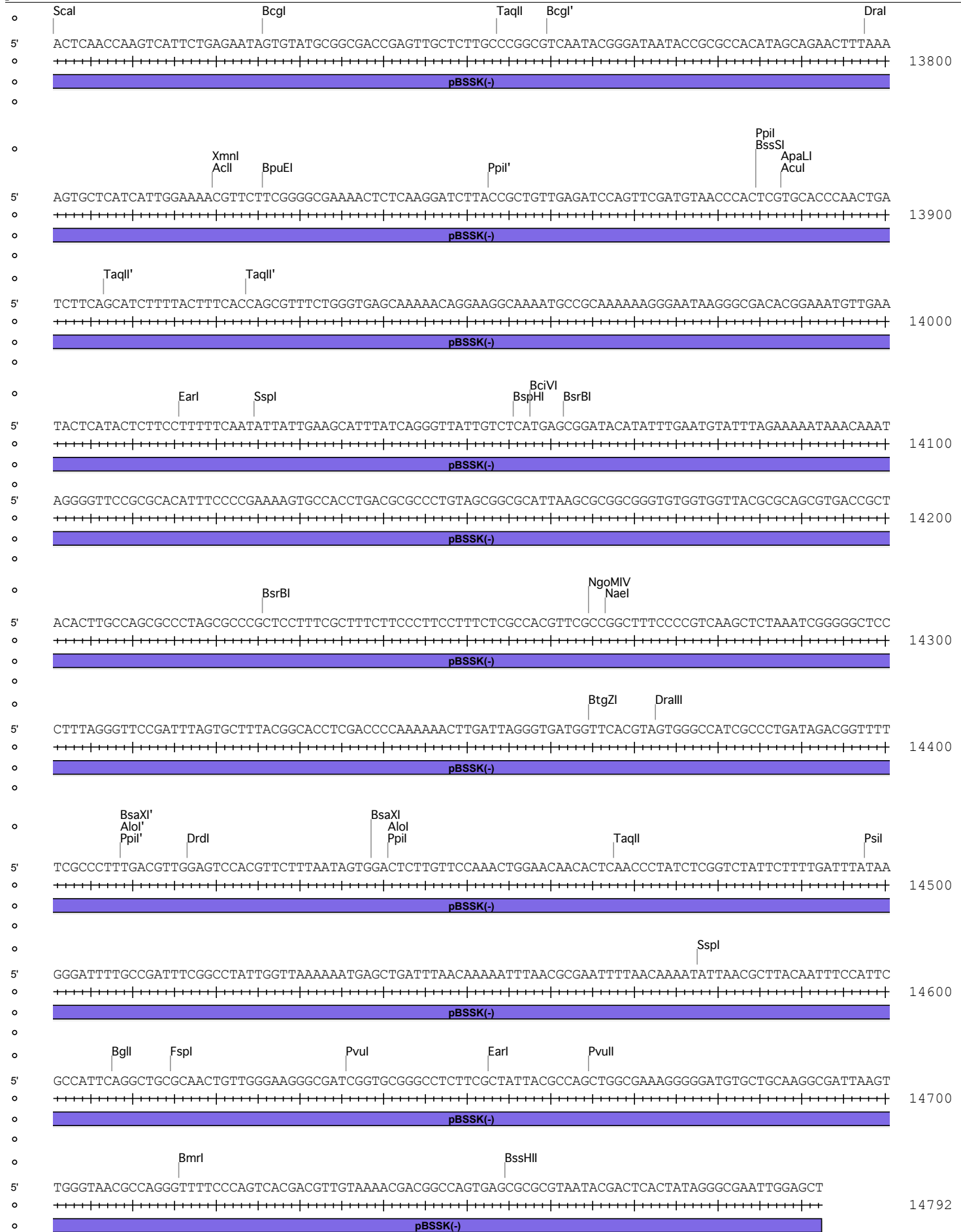

Supplement: Supplementary file 3 — Dataset S02 (PDF) [file pnas.2417252121.sd02.pdf]
